# Review of Uneven Road Surface Information Perception Methods for Suspension Preview Control

**DOI:** 10.3390/s25185884

**Published:** 2025-09-19

**Authors:** Yujie Shen, Kai Jing, Kecheng Sun, Changning Liu, Yi Yang, Yanling Liu

**Affiliations:** 1 Automotive Engineering Research Institute, Jiangsu University, Zhenjiang 212013, China; shenyujie@ujs.edu.cn (Y.S.); jkai010918@163.com (K.J.); 2 School of Automotive and Traffic Engineering, Jiangsu University, Zhenjiang 212013, China; lcn@ujs.edu.cn (C.L.); liuyl@ujs.edu.cn (Y.L.); 3 Department of Civil and Environmental Engineering, The Hong Kong Polytechnic University, Kowloon, Hong Kong 999077, China; yyi.yang@polyu.edu.hk

**Keywords:** road surface information detection, suspension preview system, machine vision, deep learning, multi-modal fusion, depth information

## Abstract

Accurate detection of road surface information is crucial for enhancing vehicle driving safety and ride comfort. To overcome the limitation that traditional suspension systems struggle to respond to road excitations in real time due to time delays in signal acquisition and control, suspension preview control technology has attracted significant attention for its proactive adjustment capability, with efficient road surface information perception being a critical prerequisite for its implementation. This paper systematically reviews road surface information detection technologies for suspension preview, focusing on the identification of potholes and speed bumps. Firstly, it summarizes relevant publicly available datasets. Secondly, it sorts out mainstream detection methods, including traditional dynamic methods, 2D image processing, 3D point cloud analysis, machine/deep learning methods, and multi-sensor fusion methods, while comparing their applicable scenarios and evaluation metrics. Furthermore, it emphasizes the core role of elevation information (e.g., pothole depth, speed bump height) in suspension preview control and summarizes elevation reconstruction technologies based on LiDAR, stereo vision, and multi-modal fusion. Finally, it prospects future research directions such as optimizing robustness, improving real-time performance, and reducing labeling costs. This review provides technical references for enhancing the accuracy of road surface information detection and the control efficiency of suspension preview systems, and it is of great significance for promoting the development of intelligent chassis.

## 1. Introduction

With societal development and technological advancement, automobiles have become an indispensable means of transportation in people’s lives [1], and demands for their overall performance are increasing daily [2]. As a critical component of automobiles [3], the suspension system significantly enhances ride comfort and handling stability by absorbing and mitigating the impacts of vertical vibration [4] and longitudinal vibration [5] caused by road unevenness [6,7,8]. Currently, suspension systems can be classified into passive suspensions [9], semi-active suspensions [10,11], and active suspensions [12]. Compared with passive suspensions (where damping and stiffness are non-adjustable), semi-active suspensions use accelerometers and displacement sensors to measure the relative motion between sprung and unsprung masses, thereby sensing road excitation [13,14]. They achieve damping adjustment via a feedback controller. Active suspensions further employ electric motors, pneumatics, or hydraulics to actively output force and dynamically adjust suspension stiffness and damping [15]. Although semi-active and active suspensions improve performance to some extent [16], they fail to achieve ideal control due to time delays in signal acquisition and output [17]. Therefore, mitigating the impact of these delays has become an urgent challenge for suspension systems [18].

In recent years, with the advancement of machine vision technology, new approaches have offered solutions to address time delay issues [19]. The application of binocular vision and LiDAR in road surface recognition has laid a reliable perceptual foundation for suspension preview control algorithms [20]. Onboard sensors detect road surface feature information in real time, enabling the acquisition of elevation data for abnormal road conditions (e.g., potholes, speed bumps). This allows the suspension system to predict upcoming road information and adjust necessary parameters promptly, effectively mitigating the impact of time delays. This technology not only optimizes the active control strategy of vehicle suspension systems to reduce suspension shocks caused by road excitation, thereby enhancing vehicle safety, passenger comfort, and handling stability [21,22,23], but also plays a significant role in preventing vehicle structural damage and reducing accident risks [24]. For example, Jaguar Land Rover has developed a warning system that alerts drivers to pothole locations [25]. ClearMotion has designed an intelligent suspension system to absorb and mitigate shocks and vibrations induced by road irregularities [26]. These innovations underscore the critical role of efficient road surface information detection for suspension preview control [27]. Consequently, advancements in suspension preview systems will effectively enhance vehicle safety and ride comfort on uneven roads while reducing economic and environmental costs [28].

Uneven road surfaces, typically characterized by significant geometric irregularities, have been shown to significantly increase vehicle fuel consumption, shorten vehicle operational lifespans, and raise maintenance costs, thereby substantially undermining the economic efficiency of vehicles [29]. Furthermore, such road conditions contribute to increased CO_2_ emissions and inefficient energy use, negatively impacting environmental sustainability and resource conservation [30]. Uneven road surfaces, exemplified by potholes and speed bumps, can exert significant impacts on vehicle suspension systems. Potholes are defined as depressions on the road surface, typically caused by material fatigue, water penetration, and excessive loads in road structural layers [31]. Speed bumps are defined as transverse raised structures in road engineering design, functioning as traffic safety measures to mandate vehicle deceleration [32]. The presence of these road surface abnormalities has become a key factor affecting vehicle ride comfort and driving safety [33].

After recognizing that road excitation negatively impacts the suspension system, perceiving road conditions in advance has emerged as a key technology for implementing vehicle suspension preview systems [34]. Traditional manual road condition inspection is the primary method for detecting road defects. While it can identify typical features of potholes and speed bumps based on engineering experience, its detection efficiency is constrained by the sampling limitations and subjective judgment variations inherent in manual inspection [35]. With the advancement of intelligent connected vehicle (ICV) technology, this manual experience-dependent detection mode can no longer meet the requirements for high-precision map construction and real-time road information perception [36]. In contrast, road information perception technology based on multi-sensor fusion enables continuous collection of road information. By integrating onboard sensors such as inertial measurement units and visual perception modules, it provides a forward-looking preview of road excitation for the suspension system [37].

**Figure 1:**
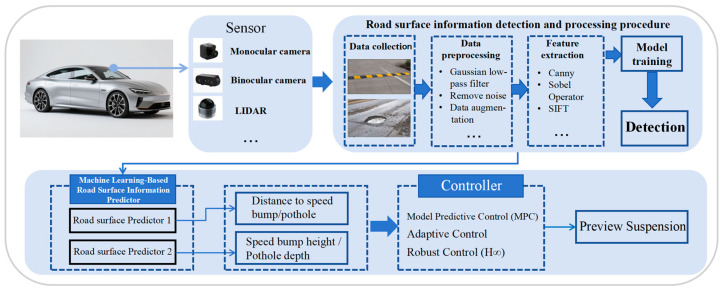
Suspension preview system based on machine vision.

The current machine vision-based suspension preview system adopts a general framework in Figure 1 comprising a sensor module, an image processing module, a detection module, and a control module. Sensors mounted on the vehicle front acquire information on upcoming road features (e.g., potholes, speed bumps). The image processing module processes the collected data. The detection module trains a model using the processed data, then applies this model to detect road surface features from sensor-captured road information, aiming to obtain speed bump/pothole depth and vehicle-to-target distances. The control module converts the detected depth and distance information into target suspension preview damping and stiffness, thereby implementing suspension preview control.

This article summarizes technologies for detecting uneven road surface conditions, which can effectively enhance the accuracy of road information detection. It comprehensively outlines detection methods for road surface conditions primarily involving potholes and speed bumps, classifying them into five primary categories: traditional dynamic methods, traditional two-dimensional (2D) image processing, three-dimensional (3D) point cloud processing, machine vision/deep learning methods, and multi-sensor fusion methods. Based on vehicle dynamics, inertial sensors are used to collect vehicle body vibration signals, which are combined with time-domain analysis [38] and frequency-domain analysis [39] for road information detection. Time-domain analysis employs peak detection algorithms combined with vibration duration characteristics for preliminary discrimination; frequency-domain analysis uses classical methods such as wavelet transform [40] and fast Fourier transform (FFT) [41]. The 2D methods for road surface information detection primarily include thresholding, damaged area identification, and edge extraction [42]. Using histogram-based thresholding [43], Otsu thresholding [44], morphological closing operations [45], and spectral clustering [46], these methods extract potential pothole contours from damaged areas, enabling accurate detection of road surface information. Three-dimensional point cloud technology enables reconstruction of road surface 3D point clouds via LiDAR or stereo vision [47]. These methods can obtain 3D contour information of road irregularities such as potholes and speed bumps, facilitating better adjustment of suspension parameters to reduce suspension impacts caused by road excitation [48]. The rapid advancement of machine learning and deep learning has revolutionized road information detection methods. For instance, models such as CNN [49], U-Net [50], and YOLO [51] rely on large road datasets and rapid, accurate, and automatic road information detection is achieved through image classification [52], object detection [53], and semantic segmentation [54]. Finally, multi-sensor fusion methods utilize multi-modal data input to compensate for the inaccuracies and poor robustness of single sensors. For example, fusing multi-modal data (e.g., 2D images and 3D point clouds) with machine/deep learning technologies can enhance the accuracy and robustness of road information detection, laying a foundation for the suspension preview systems of autonomous vehicles [55]. Based on road surface information detection, accurately acquiring elevation data of depressions or protrusions on uneven roads has become critical for better utilizing such information and enhancing the control accuracy of suspension systems [56].

This article systematically reviews technological advancements in pavement information detection, aiming to provide researchers in related fields with an objective technical panoramic analysis. It lists open-access datasets for pavement potholes and speed bumps, and emphasizes the crucial supporting role of pavement elevation information acquisition in suspension preview control systems. Specifically, the contributions of this review are as follows: (1) This review focuses on the importance of forward pavement information perception in suspension preview systems. It categorizes pavement information detection methods into five categories: vehicle dynamics methods, 2D image processing, 3D point cloud analysis, deep/machine learning methods, and multi-sensor fusion methods. Additionally, it assesses the effectiveness and limitations of these methods in pavement information perception in terms of accuracy, reliability, real-time performance, and cost. (2) This review focuses on research related to pavement information detection, with particular emphasis on detection methods for potholes and speed bumps. Unlike other focused reviews, it further enriches the application scenarios of suspension preview systems. (3) This review emphasizes the importance of elevation information acquisition in pavement information detection for suspension preview systems, and synthesizes the relevant literature on pavement elevation information acquisition. (4) Finally, this review analyzes future research directions and unresolved issues in pavement information detection to further advance the development of suspension systems.

The structure of this article is as follows: Section 2 discusses potholes, speed bumps, relevant datasets, and data collection sensor technologies. Section 3 reviews various pavement information detection methods, including traditional dynamic methods, 2D image processing, 3D data analysis, machine/deep learning methods, and multi-sensor fusion methods. Section 4 synthesizes the literature focused on pavement elevation information detection. Section 5 discusses and prospects the room for improvement in pavement information detection algorithms, as well as their future applications in suspension preview systems. Lastly, Section 6 summarizes the entire study.

## 2. Collection of Road Surface Information Datasets

**Figure 2:**
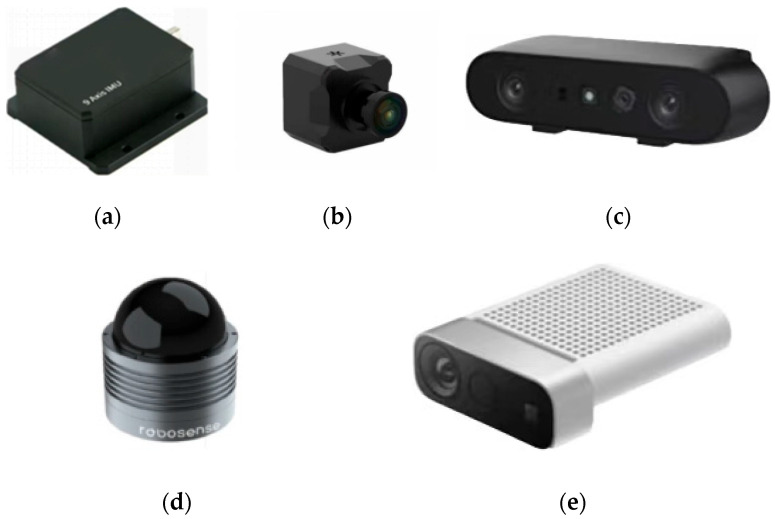
Sensors: (**a**) Inertial sensor. (**b**) Monocular camera. (**c**) Binocular camera. (**d**) LiDAR. (**e**) Depth-sensing camera.

Datasets of road surface potholes and speed bumps play a crucial role in research related to uneven road surface information detection. Current data collection systems for uneven roads typically include inertial sensors (composed of triaxial accelerometers and gyroscopes) for collecting acceleration data, monocular and binocular cameras for acquiring images, LiDAR equipment for collecting road point clouds, and specialized devices for gathering road information [57]. Typical sensors are illustrated in Figure 2.

Initially, inertial sensing elements were used to collect vibration signals for analysis and to detect road irregularities. However, this method has disadvantages such as high cost, poor real-time performance, and low efficiency [58]. Then, 2D imaging technology was employed, but it also has limitations, including the inability to accurately measure 3D contour information and susceptibility to factors such as lighting conditions, which can affect detection accuracy and reduce efficiency [59].

Subsequently, researchers turned to 3D imaging technology, which can obtain spatial geometric information of detection targets, as well as 3D contour information and surface topography of road surfaces [60]. For example, LiDAR [61], stereo vision [62], and Kinect cameras [63] have been employed. LiDAR generates high-precision point clouds by emitting laser pulses and calculating time of flight (ToF), making it suitable for large-scale elevation reconstruction of road surfaces [64]. Stereo vision relies on stereo matching algorithms between multi-view images to restore 3D coordinates, such as SIFT feature matching [65]. It offers advantages including low hardware cost and easy deployment, making it suitable for road dataset collection. The Microsoft Kinect sensor is a depth-sensing camera equipped with an RGB camera, an infrared sensor, and motion tracking capabilities, which can also be used for road imaging. However, it may produce inaccurate readings due to challenges such as infrared saturation caused by direct sunlight [66].

**Figure 3:**
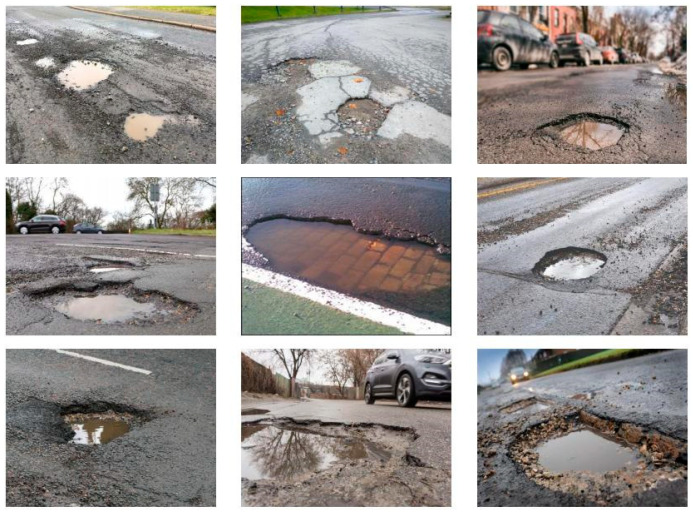
Schematic diagram of the pothole dataset.

In previous studies, several open-access datasets of potholes have been developed to support the advancement of pothole detection algorithms. For example, Dib’s dataset provides an annotated dataset of flooded and dry depressions for deep learning applications, and is the first dataset to include both flooded and dry conditions. It comprises 743 high-quality images for training and validation, with 80% used for training (570 images) and 20% for testing (173 images). This dataset was collected via image acquisition devices from different angles and is publicly available on Mendeley. The PODS dataset includes 569 images, which were augmented to 1363 images for pothole detection. These images are divided into three subsets, training (1191 images), validation (113 images), and testing (59 images), and can be accessed on Universe. Additionally, Abhinva Kulshreshth’s dataset, which combines data sourced from Google and Kaggle, contains normal and pothole images. It is divided into training (1167 images), validation (108 images), and testing (136 images) subsets, and is publicly available on Kaggle. The Pothole-600 dataset [67] provides disparity images of road potholes, captured using ZED stereo cameras. It is divided into training (239 images), validation (179 images), and testing (179 images) subsets, and is available on Google’s public platform. Furthermore, a dataset focused on semantic segmentation of potholes and cracks provides 4340 images for training, validation, and testing [68]. This dataset is split into training (3340 images), validation (496 images), and testing (504 images) subsets, accounting for 77%, 11%, and 12% of the total dataset, respectively. It can be accessed via deep learning websites. Finally, an open dataset from Japan uses in-vehicle smartphones to capture 15,435 road damage instances, including 9053 road images. It is divided into training (7718 images), testing (4630 images), and validation (3087 images) subsets, and is publicly available on GitHub. Figure 3 presents a schematic of the pothole datasets. Table 1 summarizes details of the public pothole datasets discussed in this paper.

**Figure 4:**
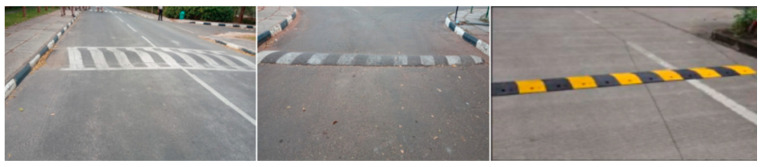
Schematic diagram of the speed bump dataset.

The number of speed bump datasets is significantly smaller than that of pothole datasets, with most being self-constructed and not publicly accessible. The “SpeedHump/bump Dataset” includes both labeled and unlabeled speed bump images, with 543 images of varying sizes and a total storage space of 88.3 MB. It is also publicly available on Mendeley. Siddharth Nahar’s dataset, named “Marked Speed Bump/Speed Breaker Dataset (India)”, contains 969 cropped and labeled images of speed bumps/breakers, with the remaining images sourced from the Internet. This dataset is also publicly available on Mendeley. Furthermore, ZIYA has released a dataset on Kaggle containing speed bumps, cracks, potholes, and normal roads, where each image represents a road segment with specific characteristics and includes various road conditions. The Universe Speed Bump Dataset (4) is used for object detection, comprising 519 color images as the training set, 49 as the validation set, and 25 as the test set. It has been applied to several YOLO models, such as YOLOv5, YOLOv7, YOLOv8, and YOLOv9, and is available on the Universe website. Also available on the Universe website is one of the largest datasets in this field—the Universe Speed Bump Dataset (15)—which is used for object detection and includes 1014 training images, 339 validation images, and 339 test images. Figure 4 presents a schematic of the speed bump datasets. Table 2 summarizes details of the public speed bump-related datasets discussed in this paper.

## 3. Method Classification

Early researchers used traditional dynamic methods (e.g., accelerometers [69], gyroscopes [70], and GNSS [71,72]) to detect and locate potholes and speed bumps on road surfaces. They collected vehicle body vibration signals and utilized wavelet transform, Kalman filtering, or fast Fourier transform for denoising and feature extraction [73,74]. However, due to the limitations of traditional dynamic analysis methods in distinguishing road anomalies with similar vibration waveform characteristics, these methods may confuse the high-frequency features of potholes and speed bumps. Consequently, the suspension preview system has difficulty providing complete road information in advance. Subsequently, computer vision methods were applied to the detection of pavement potholes and speed bumps. Based on technical approaches, they can be roughly categorized into three types: 2D image processing, 3D point cloud analysis, and machine/deep learning. Two-dimensional image processing uses single cameras or other acquisition devices to obtain RGB images of road surfaces. Traditional visual algorithms (e.g., edge detection [75] and morphological operations [45]), shallow machine learning (e.g., SVM [76] and random forest [77]), thresholding, and segmentation are employed to detect information about uneven road surfaces. Another approach involves 3D road point cloud technology or stereo vision reconstruction technology. This method can fit planar or quadratic surfaces to observed road point clouds and, through analysis, obtain 3D contour information of the uneven [78]. In contrast, machine/deep learning-based methods employ advanced CNN [79] and YOLO [80,81], using object detection, semantic segmentation, and image classification to detect uneven road information, achieving better results. Additionally, multi-sensor fusion methods enhance system robustness through sensor information fusion or the combination of multiple algorithms [82]. By integrating multi-source perception information with machine/deep learning, these models effectively address challenges posed by complex road conditions, shadows, or overexposure. The combination of these methods enables rapid and accurate detection of target objects and can also obtain valuable 3D contour information by integrating multi-source data [83]. Thus, multi-sensor fusion methods offer better robustness and accuracy.

### 3.1. Road Surface Information Detection Based on Traditional Dynamics

**Figure 5:**
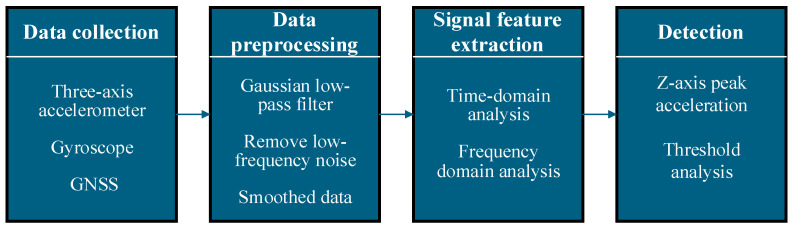
Flowchart of Vehicle Dynamics Road Surface Information Detection Process.

Traditional dynamic methods for pavement information detection primarily identify pavement characteristics by analyzing vehicle vibration signals. Their typical process includes three steps: first, a Gaussian low-pass filter is used to remove low-frequency noise and smooth data; second, wavelet transform or fast Fourier transform is applied to decompose vibration signals and extract frequency-domain features; finally, algorithms such as Z-Thresh [84] and Z-Diff [85] are used to detect pavement characteristics. Figure 5 illustrates the processing flow of pavement information detection for traditional dynamic methods.

Mednis et al. (2011) [86] proposed a real-time pothole detection system based on accelerometers. They compared four methods: Z-THRESH (*Z*-axis acceleration threshold method), Z-DIFF (*Z*-axis acceleration difference method), STDEV (*Z*-axis standard deviation method), and G-ZERO (three-axis near-zero feature method). Using multi-brand vehicle data acquisition equipment, they conducted an accuracy experiment on urban roads in Latvia. Actual measurement results showed that the Z-DIFF algorithm exhibited the best comprehensive performance, with a true positive rate of 92%. Specifically, the detection rate for large potholes was 100%, while those for small potholes and pothole clusters were 89% and 90%, respectively.

Wang et al. (2015) [87] proposed a pothole detection method based on mobile sensing. They innovatively integrated the Z-THRESH and G-ZERO algorithms, calculated and normalized multi-axis acceleration data using Euler angles to eliminate device angle constraints, and adopted spatial interpolation to reduce GPS positioning errors. Experimental results showed that this method achieved 100% accuracy in 10 tests with no false positives, and the positioning error was reduced from 1747 m to 11.74 m. However, this method has limitations, including a small sample size and failure to consider energy optimization for mobile devices. Future work should involve expanding the data scale and developing low-power algorithms.

Rishiwal and Khan (2016) [88] proposed a vibration-based automatic detection method for identifying speed bumps and potholes. This method detects speed bumps by analyzing sudden changes in *z*-axis acceleration during vehicle travel and assesses their severity through threshold classification. Experiments were conducted on a 4 km flat road, and when compared with manual detection, it achieved an accuracy rate of 93.75%. However, this method does not fully consider the impact of vehicle speed changes on thresholds and depends on the fixed placement of sensors.

Aljaafreh et al. (2017) [89] proposed a method for detecting speed bumps by combining a fuzzy inference system (FIS) with accelerometers. This fuzzy inference system detects and identifies speed bumps based on vehicle vertical acceleration and speed changes. In the experiment, a smartphone was installed on the car chassis, and data were collected at different speeds. Results showed that the system could effectively distinguish speed bump vibrations from normal road fluctuations but exhibited detection errors at high speeds. The innovation of this method lies in using fuzzy logic to handle the uncertainty of sensor data, overcoming the insufficient sensitivity of traditional threshold methods to dynamic speed changes.

Pooja and Hariharan (2017) [90] developed a real-time speed bump detection system based on crowdsourced data, using smartphone accelerometers and GPS for data collection. This method employs a Gaussian low-pass filter to reduce noise and smooth data, and dynamically adjusts thresholds based on vehicle speed. Test data were analyzed using the SMO classifier, achieving 93.6% accuracy. Experiments revealed that speed bumps correspond to sudden positive increases in *z*-axis acceleration, while potholes correspond to sudden negative increases.

Celaya-Padilla et al. (2018) [91] proposed a deceleration zone detection method based on acceleration features. To collect multi-dimensional dynamic data during vehicle operation, this study integrated accelerometers, gyroscopes, and GPS sensors. Using a Genetic Algorithm (GALGO) for feature selection and model optimization, they established a logistic regression classification model. Experiments were conducted in complex road environments. In blind tests, this model achieved an accuracy of 97.14%, an AUC of 0.9784, and a false alarm rate below 0.018%, thereby demonstrating significantly superior performance to traditional threshold-based methods.

Rodrigues et al. (2019) [92] proposed a real-time pothole detection method based on Haar wavelet transform. This method analyzes the *z*-axis signal of the vehicle accelerometer, extracts high-frequency features using Haar wavelet decomposition, and employs two-step thresholds to analyze wavelet coefficients. It offers the advantage of low-cost processing in both signal acquisition and analysis stages. The algorithm’s effectiveness was verified through experiments on robot-simulated roads and real campus bridge scenarios, which successfully distinguished normal road fluctuations from pothole impacts. Its advantages include not requiring manual threshold calibration and having high computational efficiency (processing time per frame is less than 10 ms), making it suitable for large-scale deployment. This method is effective in both controlled and real scenarios.

Lekshmipathy et al. (2021) [93] proposed a new pothole detection method based on acceleration sensors. To address the limitations of single algorithms under varying vehicle speeds and pothole sizes, the authors proposed combining the z-peak, z-sustainable, z-x, z-diff, and standard deviation algorithms, and enhancing detection accuracy via sensor redirection and data preprocessing. The study calibrated data collected by an external triaxial accelerometer using external reference data and validated detection results using synchronous video acquisition. Experimental results showed that using the optimal threshold combination (2 g, 0.45 g, 1.5 g/0.5 g, 2 g, 1.4 g) achieved a true positive rate of 93.18% and a false positive rate of 20%, markedly improving pothole detection efficiency.

Kalushkov et al. (2021) [94] proposed using two *z*-axis-based detection algorithms (Z-THRESH and Z-DIFF), utilizing built-in smartphone accelerometers and data from the external GY-521 accelerometer to determine the number and location of potholes. By setting a threshold of 0.3 g, the system’s accuracy in detecting large potholes significantly improved, with the true positive rate reaching 86.66%.

Cao et al. (2024) [95] proposed a speed bumps detection system based on vehicular networks. This system identifies speed barriers by analyzing vibration frequency characteristics derived from the speed difference between the front and rear wheels, and employs continuous wavelet transform (CWT) for feature extraction. The system comprises two modules: the onboard detection unit and the cloud-based data fusion platform. The onboard module caches 20 s of wheel speed and GPS data, and uploads detection results to the cloud every 10 s for centralized processing. Experiments were conducted using ROS data packets based on real-world data simulating actual driving scenarios. In an 11 min test, the system achieved 100% detection accuracy with no false alarms. This design renders the system suitable for large-scale vehicular networks and aligns with the development trend of connected vehicle technology.

Yin et al. (2024) [96] utilized accelerometers, GPS sensors, and extract data features filter (EDFF) to detect road speed bumps and potholes. For acceleration data preprocessing, this method employed Euler points, the least squares method, and wavelet technology. The genetic algorithm was then used to identify parameters of the vehicle model. Experiments using real-world data from Harbin showed that speed bump detection accuracy reached 100%, while pothole detection accuracy was 75%. This method is practical and cost-effective for inspecting speed bumps and potholes on low-grade roads. Future work could focus on detecting small potholes under high-speed conditions.

Zhang et al. (2025) [97] proposed a method for identifying potholes and determining their depths based on vibration acceleration. The study first preprocessed collected acceleration data; then established the vibration equation, solved it using the Newmark method to obtain vehicle acceleration when passing over potholes, and simultaneously identified vehicle vibration parameters through drop tests; finally, the particle swarm optimization algorithm was used to estimate pothole depths based on vibration acceleration, with verification tests conducted. Results showed that the proposed pothole identification algorithm, based on vibration acceleration thresholds, could accurately identify potholes, with an average error rate of 8.94% and a minimum error rate of only 0.50%. Table 3 summarizes studies using traditional dynamic methods.

The traditional dynamic method detects road characteristics by analyzing vertical vibration signals of the vehicle body. Common algorithms for pothole detection include Z-THRESH, Z-DIFF, STDEV (Z), and G-ZERO. These are supplemented by wavelet transformation (e.g., Haar wavelet) to extract high-frequency impact features, or machine learning models (e.g., SVM [76], CNN-LSTM [98]) to classify time-series window features. Speed bump detection relies on identifying sudden positive increases in *Z*-axis acceleration, combined with dynamic threshold adjustment [99], fuzzy inference systems (FIS) [100], or continuous wavelet transform (CWT) [101]. Its advantages include simple deployment, low cost, sensitivity to road excitation responses, effective capture of specific high-frequency vibration characteristics caused by potholes or speed bumps, relatively mature algorithms, and ease of implementation with reliable results. However, this method can only provide road information along the current trajectory and cannot preview road conditions ahead.

### 3.2. Road Surface Information Detection Based on 2D Data

**Figure 6:**
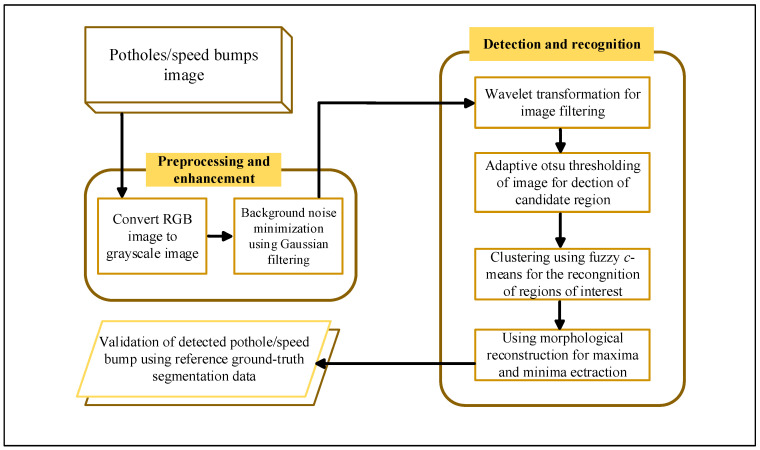
A 2D image detection method using wavelet transform and FCM (Fuzzy C-Means).

As researchers continue to explore, using 2D images to detect road information has proven effective. Compared to traditional vehicle dynamics methods, 2D image-based methods can depict road information outlines. This approach involves grayscale processing and binarization of input 2D images to reduce computational complexity. It also employs techniques such as Canny edge detection [102], Otsu thresholding [44], gray-scale road image segmentation via thresholding [43], and Hough transform [103] to segment and extract damaged areas. In subsequent processing, morphological closing operations and median filtering can reduce noise [104]. Finally, intensity histogram data can identify road surface information. Figure 6 illustrates A 2D image detection method using wavelet transform and FCM (Fuzzy C-Means).

Buza et al. (2013) [105] proposed an unsupervised vision-based pothole detection method. Unlike other methods, it requires no expensive equipment, complex filtering, or training stages. Its core process involves Otsu thresholding-based image processing and spectral clustering: after detecting defect-containing image frames, spectral clustering analyzes grayscale image histogram data to identify pothole areas. This method enables pothole detection using low-cost equipment and rough surface estimation, offering cost-effectiveness advantages. Experiments on 50 distinct pothole images showed its surface estimation accuracy reached 81%.

Kieran and Mullari (2014) [106] proposed a method to detect speed bumps in a single road image and automatically generate a three-dimensional view. This method segments the road area using Canny edge detection and the Hough transform to estimate the vanishing point, locates speed bumps via morphological dilation and erosion operations, and calculates the distance between the actual ground position and speed bumps using Euclidean distance. Experimental results show that the depth error between manual measurements and algorithmic calculations is only 4.375%. The method performed stably in 400 image-based tests but was only applicable to scenarios with a single vanishing point.

Ryu et al. (2015) [107] proposed a pavement pothole detection method based on 2D image features. First, the method uses histogram threshold segmentation and morphological closing operations to extract dark areas in the image. Second, it screens candidate regions based on geometric features. Finally, it classifies candidate regions as potholes by comparing pothole and background features. Experiments were conducted using 30 images collected from the Korean National Highway, showing that this method achieved an accuracy of 91.0%, with precision and recall of 85.3% and 93.5%, respectively, and was significantly superior to the traditional texture standard deviation-based method (71.6% accuracy). By integrating multiple features, this method reduced the false detection rate but was limited by complex shadow scenes and overlapping dark areas.

Devapriya et al. (2015) [108] proposed a speed bump detection method based on image processing. First, it converts RGB images to grayscale to reduce computational complexity, then converts grayscale images to binary images to remove noise. Second, it uses morphological operations to extract speed bump regions. Third, it identifies the peak position of speed bumps via horizontal projection analysis and determines their locations. This method achieved an average detection accuracy of 84.5% for speed bumps with appropriate markings. By optimizing image processing (15 fps) and employing a pure visual solution without external hardware, it can run in real time on mainstream vehicle processors, providing a low-cost road anomaly detection solution for autonomous driving systems.

Schiopu et al. (2016) [109] developed a low-cost method for detecting and tracking potholes based on onboard video. The method first selects the region of interest (ROI), performs threshold segmentation to generate candidate regions, then extracts features (e.g., size, surface regularity, contrast, and contour shape) from each region, and uses a decision tree to distinguish shadows from potholes. Finally, cross-frame tracking is achieved by combining Euclidean distance mapping. Experiments conducted using 34 min of high-definition video successfully detected 55 potholes (accuracy: 90%) with 6 false detections (mainly due to edge shadows). The algorithm exhibits high computational efficiency (0.1–0.2 milliseconds per frame), is suitable for crowdsourced road detection, but its tracking stability in highly dynamic scenes requires optimization.

Devapriya et al. (2016) [110] proposed an image processing framework based on Gaussian filtering, median filtering, and connected domain analysis. Through preprocessing to enhance speed bump characteristics and combining binarization with morphological operations, it achieves road marking detection. The study focused on diverse speed bump types in India, with a 90% detection rate for clearly marked ones and only 30% for unmarked types. The Raspberry Pi-based real-time processing system can operate independently with low network dependence. Its low algorithmic complexity makes it suitable for resource-constrained devices, but it relies on intact road markings, and its performance deteriorates significantly at night or in adverse weather.

Ouma and Habn (2017) [111] proposed a method for detecting asphalt pavement potholes based on 2D color images. This method integrates wavelet transform, fuzzy c-means (FCM), and morphological reconstruction techniques. It first filters and denoises images via multi-scale wavelet transform and extracts texture features; then uses the FCM algorithm to cluster images, distinguishing pothole areas from non-pothole areas; finally, optimizes pothole edge detection through morphological reconstruction. Experiments verifying 75 road images showed an average Dice similarity coefficient of 87.5%, a Jaccard index of 77.7%, and a sensitivity of 97.6% in pothole detection, with an average pothole area extraction error of 8.5% and a standard deviation of 4.9%. The study indicates this method can effectively detect early-stage potholes at low cost.

Srimongkon and Chiracharit (2017) [112] proposed a method for detecting speed bumps using the Gaussian Mixture Model (GMM). This method separates speed bumps from the road and surrounding environments based on their stripe features and eliminates noise via morphological closing operations. Experiments were conducted in both daytime and nighttime: daytime detection accuracy was 94.7%, dropping to 70.8% at nighttime, indicating the algorithm is sensitive to lighting changes and occlusion.

Wang et al. (2017) [113] proposed an innovative method based on the wavelet energy field and Markov Random Field (MRF). This method constructs a multi-scale wavelet energy field, integrates gray difference and texture features, and combines morphological processing and geometric criteria to achieve pothole detection. Subsequently, using the energy field as the label field and the original image as the feature field, it optimizes segmentation via the MRF model and enhances edge extraction through morphological operations. Experiments using 120 road surface images (90 for testing) showed a detection accuracy of 86.7%, precision of 83.3%, and recall of 87.5%. However, it is sensitive to internally smooth or bright potholes and easily interfered with by mesh-like shadows and dense cracks.

Chung and Khan (2019) [114] proposed a real-time image processing technique based on the watershed algorithm to detect multiple potholes in asphalt pavements. To address limitations of existing methods, such as high cost, long processing time, or insufficient environmental adaptability, they proposed an improved algorithm: First, it extracts features via inverse binary processing and Otsu threshold segmentation, combining them with morphological opening and closing operations for noise reduction and edge enhancement. Then, it locates marker points using distance transformation, and finally segments pothole areas via the watershed algorithm. Experimental results show this algorithm can effectively detect multi-scale potholes on smooth and deteriorated road surfaces, with an average processing speed of 33.1 fps, thus meeting real-time requirements.

Sirbu et al. (2021) [115] proposed a real-time speed bump detection algorithm based on a single camera. This method takes frames from a single camera as input and converts them into grayscale images to reduce processing requirements. First, it smooths the image using a Gaussian filter, then determines the road area as the region of interest (ROI) via semantic segmentation. It combines the improved ED Line algorithm to obtain edge lines of irregular roads. Tests on the internal dataset show it achieved an F1 score of 0.783 and an IoU of 0.533, with the algorithm performing well in detecting speed bumps obscured by vehicles. Table 4 summarizes studies employing 2D methods.

This method identifies road information by analyzing road images. It mainly employs the Otsu thresholding method and histogram-based algorithms to detect and segment damaged areas. Additionally, the Canny edge detection algorithm can more clearly highlight the boundaries of potholes/speed bumps. During image processing, techniques such as Gaussian filtering, median filtering, and morphological processing can be used to reduce noise. Based on 2D image processing methods, which are characterized by low cost and easy deployment [116], it can provide forward road preview information. However, it heavily relies on lighting and weather conditions, which affect the segmentation accuracy of grayscale and color images and reduce detection accuracy [117]. Moreover, 2D images cannot provide three-dimensional information, restricting the performance of suspension preview control. Thus, further research on methods for constructing three-dimensional contours is required.

### 3.3. Road Surface Information Detection Based on 3D

**Figure 7:**
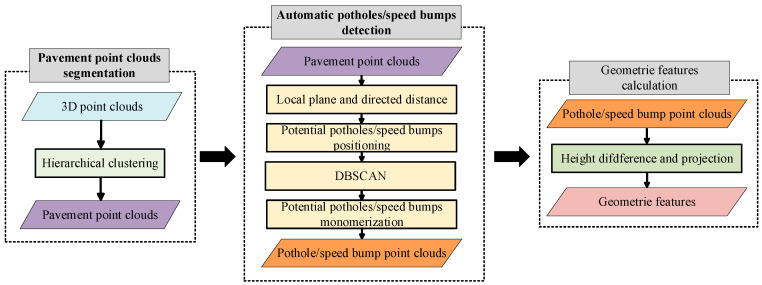
Detection flowchart using 3D point cloud segmentation.

With the development of environmental sensing devices, three-dimensional environment construction, due to its ability to provide valuable three-dimensional contour information, is gradually becoming a promising method in the field of road information detection [118]. Currently, mainstream 3D reconstruction methods are mainly based on multi-view and point cloud information. The multi-view method utilizes a collection of 2D images captured from multiple viewpoints to represent 3D geometric shapes in a simple way, but some detailed geometric information may be lost during the projection process [119]. In contrast, point clouds are a set of points in 3D space, each consisting of 3D coordinates (*x*, *y*, *z*) and other information. The denser the point cloud, the higher the accuracy of the reconstructed model. Point cloud data can typically be obtained via 3D scanning devices or LiDAR [120]. When processing 3D road point cloud data, the first step is to use the RANSAC plane fitting region-growing algorithm to separate road point clouds from non-ground objects (such as vehicles and pedestrians). Then, the processed point cloud data are segmented. By comparing and analyzing the fitted point cloud with the observed point cloud, more detailed three-dimensional contour information can be obtained [121]. Figure 7 shows detection flowchart using 3D point cloud segmentation.

Fernandez et al. (2012) [122] proposed a real-time free space and speed bump detection system for urban autonomous driving applications. This system integrates low-cost LiDAR and cameras, obtaining sensor extrinsic parameters through offline calibration. The LiDAR detects road slope changes by analyzing four-layer horizontal scanning data, while the camera verifies speed bumps based on recognizing white stripe features. The algorithm compensates for vehicle movement by integrating laser data and extracts candidate curb points using the angle threshold method. Experimental results show that at a speed of 40 km/h, the system can detect speed bumps 12 m in advance, with a 100% detection accuracy and a processing speed of 154 FPS, meeting real-time requirements.

Moazzam et al. (2013) [123] proposed a low-cost method for measuring and visualizing road surface potholes based on Microsoft Kinect sensors. This method uses the infrared camera of Kinect to capture depth images, realizes intuitive visualization of potholes by generating a three-dimensional grid, and combines the trapezoidal integration method to calculate the volume, area, and geometric parameters of potholes. Experimental results show that through local minimum value analysis and coordinate transformation algorithms, this method can accurately extract pothole features, with an average depth error of ±15%. Compared with traditional stereo vision and LiDAR technologies, Kinect has the advantages of low cost and the ability to directly obtain depth information without complex calculations, but its measurement range (0.8–3.5 m) is limited.

Melo et al. (2018) [124] developed a road speed bump detection system based on 24 GHz interferometric radar and stepped-frequency continuous wave (SFCW), and could estimate the height of speed bumps. By measuring the phase difference using vertical baseline interferometry and enhancing height resolution with the virtual baseline algorithm, they successfully detected speed bumps up to 8 cm high. The study utilized an indoor simulated road scenario, and results showed that the system’s height estimation error was less than 5%, verifying the reliability of millimeter-wave radar in adverse weather conditions. This research was the first to combine the 24 GHz frequency band with interferometric technology, providing a low-cost solution for real-time perception of small road obstacles by autonomous vehicles.

Tsai and Chatterjee (2018) [125] proposed a pavement pothole detection and classification method based on 3D laser technology and the watershed algorithm. This method utilizes high-precision 3D pavement data (with a lateral resolution of 1 mm), using steps such as 3D data acquisition, data correction, and water basin detection, and combines the LTPP protocol standard to achieve pothole detection. Experimental verification shows that this method achieves an accuracy of 94.97%, precision of 90.80%, and recall of 98.75% in road tests in Atlanta and Savannah. False detections mainly stem from interference by manhole covers or debris.

Lion et al. (2018) [126] proposed a method for detecting speed bumps and estimating their height based on the Microsoft Kinect sensor. This method uses the Kinect’s RGB and infrared cameras to obtain color and depth images, and combines 3D scene reconstruction technology to identify speed bumps in real time and extract their attributes (e.g., height and distance). Compared with traditional stereo vision methods, the Kinect directly provides depth maps, eliminating the need for complex camera calibration and image correction. Experimental results demonstrate that the system achieves an average accuracy of 98.93% in distance measurement to speed bumps, 86.84% in height estimation, and a maximum error of ±2.4 cm.

Wu et al. (2021) [127] proposed an efficient framework for pothole detection and tracking. This framework first uses an advanced disparity estimation algorithm, GPT-SGM (a state-of-the-art disparity estimation algorithm), to fit a quadratic surface to the three-dimensional road point cloud, employing a fast and accurate surface normal estimator, “Three-Filters-To-Normal (3F2N),” to eliminate outliers. By comparing the actual and simulated three-dimensional road point clouds, the framework extracts the pothole point cloud. To improve real-time performance, it adopts the DSST algorithm for scale-adaptive tracking in the bird’s-eye disparity map. Experiments show that this method achieves a detection accuracy of 98.7% and can effectively handle occlusion and dynamic scale changes.

Ma et al. (2023) [128] proposed an automatic road pothole detection method based on mobile laser scanning (MLS) point cloud data. To address the issue of inaccurate directional distance in the surface fitting method, this method innovatively combines directional distance with skew distribution characteristics. Firstly, the road point cloud is vertically segmented, and the local plane is fitted using RANSAC and the least squares method. Potential potholes are then singularized through DBSCAN clustering. Further, it uses the surrounding road point cloud to re-fit the plane, combining the negative skew distribution histogram and its skewness coefficient to accurately determine the pothole boundary. Experiments show that this method can effectively identify different forms of potholes under complex road conditions, with a geometric feature extraction deviation below 10%, significantly outperforming traditional baseline methods.

Fan and Chen (2023) [129] proposed a method for detecting speed bumps using mobile laser scanning (MLS) point cloud data and systematically evaluated the performance of two deep learning models (PointNet++ and PointCNN). Their results were compared with those of the traditional region-growing method. The experiments were based on 171 GB point cloud data from Trondheim, Norway, with 921 speed bump samples labeled. Results showed that deep learning methods performed comparably to traditional methods in terms of intersection over union (IoU) and recall rate, with an average IoU of approximately 0.8, but rely on large volumes of labeled data; the region-growing method detects speed bumps by identifying changes in normal vectors, requiring no training and exhibiting high adaptability.

Sun (2025) [130] proposed a method for quantifying the volume of road potholes based on 3D point clouds. The study used an RGB-D depth sensor (Azure Kinect DK) to collect data, reducing noise through voxel filtering and voxel downsampling to improve data processing efficiency; it utilizes RANSAC and Euclidean clustering to segment the point cloud; and combines the Alpha Shapes algorithm for 3D reconstruction and volume calculation. Experiments showed that under conditions of no illumination interference and an imaging distance of 110 cm, the method achieved an average accuracy of 96.4%. The study verified the efficiency of depth cameras in ideal scenarios, while also noting that factors such as water accumulation and lighting condition changes may affect measurement accuracy in practical applications. It is recommended to enhance robustness by combining LiDAR technology in the future. Table 5 summarizes studies utilizing 3D methods for detection.

3D-based methods mainly rely on road point cloud information for detection. LiDAR generates high-precision road models through point cloud matching and surface fitting; stereo vision calculates depth information via disparity maps; and depth cameras (e.g., Kinect) output real-time road elevation maps. Their advantages include the ability to pre-reconstruct spatial geometric features of the upcoming road (e.g., pothole depth, speed bump height), providing key input for suspension preview. Additionally, they are not significantly affected by light changes, suitable for nighttime use, and can effectively distinguish road shadows, textures, and actual deformations through point cloud modeling. However, the main limitations of these methods are high hardware costs, complex real-time processing requirements, and susceptibility to reconstruction failure or significant accuracy degradation in rainy and snowy weather.

### 3.4. Road Surface Information Detection Based on Deep/Machine Learning

Machine learning and deep learning have become core paradigms of data-driven technologies, widely applied across numerous fields. Machine learning can make predictions by learning patterns in data and is categorized into two approaches: supervised learning and unsupervised learning. The most significant distinction between them lies in the presence of data labels during training [131]. Unsupervised learning involves no labels in training samples or clear output results. Instead, computers autonomously learn similarities among samples to classify them [132]. Compared with supervised learning, its key advantage is minimizing the impact of human annotation on results. In the field of road information detection, image classification, semantic segmentation, and object detection [133], which are among the most common data-driven technologies and current mainstream computing technologies for visual tasks, enable more effective detection of road information.

**Figure 8:**
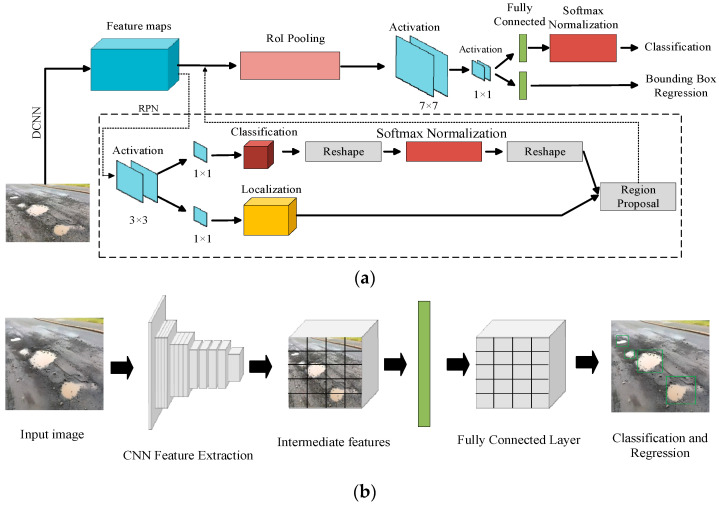
Faster R-CNN and YOLO processing workflow: (**a**) Faster R-CNN; (**b**) YOLO.

As deep learning continues to advance, mainstream models such as YOLO [134], RetinaNet [135], Faster R-CNN [136], and SSD MobileNet [137] are widely used for road information detection. At the same time, novel techniques for DCNN-based road image object detection are constantly emerging. Faster Regions with Convolutional Neural Network (Faster R-CNN) is an improved version of R-CNN. It primarily employs the Region Proposal Network (RPN) to replace R-CNN’s selective search algorithm, making it the first truly end-to-end network. Compared to R-CNN, it offers higher detection speed and accuracy. Mainstream single-stage detection methods, such as YOLO, treat detection as a regression task. They utilize an end-to-end feature extraction network to extract target features and directly apply them to bounding box classification and regression. Faster R-CNN and YOLO processing workflow is illustrated in Figure 8.

Shah and Deshmukh (2019) [138] proposed a CNN-based speed bump detection model to identify speed bumps and improve driving safety and comfort. They used ResNet-50 for image classification, optimizing training efficiency via transfer learning. Using an open dataset, it achieved an 88.9% true positive rate (TPR) after training. For target detection, YOLO combined with ResNet-50 located speed bumps. After 200 training epochs with an initial learning rate of 0.0003, the validation set showed high detection confidence. Compared with traditional sensor-based solutions, this vision-driven method provides forward-looking road preview for active suspension adjustment and has practical application potential.

Ping et al. (2020) [139] proposed a street pothole detection system based on deep learning, which uses four models for training and testing: YOLOv3, SSD, HOG combined with SVM, and Faster R-CNN. The study conducted model training and testing after preprocessing the dataset, comparing the accuracy of these models. Experimental results showed that YOLOv3 achieved the best performance in terms of speed and reliability, with a detection accuracy of 82%, making it suitable for real-time detection scenarios.

Arunpriyan et al. (2020) [140] proposed a deep learning algorithm based on SegNet for detecting speed bumps. Using a monocular camera, they collected road images under multi-lighting and multi-angle conditions, and expanded the dataset to 1302 images through data augmentation (e.g., rotation, brightness adjustment). SegNet was trained for pixel-level classification. Experiments showed that the model achieved a global accuracy of 91.78% in daytime scenes but exhibited poor detection performance for unmarked speed bumps and vertical-view images. The advantage of this method is its low-cost monocular vision solution, which can be improved by replacing the monocular camera with a stereo camera (with depth information) to enhance detection accuracy.

Gupta et al. (2020) [141] proposed a thermal imaging pothole detection and location method based on the improved ResNet50-RetinaNet model. The authors constructed a dataset consisting of 1418 thermal imaging images, covering various weather scenarios. The experiments compared the improved ResNet34-SSD and ResNet50-RetinaNet models, with detection accuracy significantly improved. Results showed that the average precision (AP) of the ResNet50-RetinaNet model reached 91.15%, outperforming ResNet34-SSD (74.93%) and existing models such as YOLOv2. This method achieved high-precision pothole detection in complex environments with support from low-cost thermal imaging equipment. The innovation of this study is the first combination of thermal imaging with the improved RetinaNet, providing a new approach for road pothole detection in low-visibility scenarios.

Patil et al. (2020) [142] proposed the application of conditional generative adversarial networks (cGAN) in the segmentation of speed bumps. Using 550 images from a public dataset (including labeled and unlabeled speed bumps), 490 of these were used for training and validation. This method takes monocular images as input and employs a GAN network to segment speed bumps. The average intersection-over-union (IoU) across various scenarios reached 93.8%, enabling effective detection of faded markings and partially obstructed speed bumps. However, the model exhibited missed detections for distant speed bumps and occasional false detections. Compared to LiDAR and traditional image processing, cGAN demonstrates strong generalization ability on small datasets, providing a new approach for low-cost visual solutions.

Dewangan and Sahu (2020) [143] addressed the issues of high cost and poor environmental adaptability of LiDAR by designing a customized CNN model: input images are preprocessed, and features are extracted via multi-scale convolutional layers to locate speed bumps, combined with bounding box regression. Using 575 original images, which were enhanced via data augmentation to 3450 images, the model achieved an accuracy of 98.54% and precision of 99.05%. Additionally, a distance estimation algorithm based on pixel changes was proposed; it utilizes the linear relationship between the width of speed bump bounding boxes and vehicle distance to achieve real-time distance measurement. Experiments demonstrated that this system can effectively identify marked and unmarked speed bumps in indoor simulation environments, with performance outperforming existing sensors and image processing methods.

Fan et al. (2021) [144] innovatively proposed the Graph Attention Layer (GAL) for semantic segmentation, which can be flexibly integrated into existing CNN architectures. The authors addressed background interference by releasing the first multi-modal road dataset (including RGB, raw disparity maps, and transformed disparity maps), where transformed disparity maps significantly enhanced the discrimination of damaged areas. Experiments compared the DeepLabv3+ model integrated with GAL with nine state-of-the-art CNN models across three training datasets. This model achieved the best pothole detection accuracy across all training datasets, verifying its efficiency and robustness. Additionally, GAL demonstrated good generalization ability in general scene datasets, with a 4% increase in mIoU and 3% in mFsc, providing a new optimization approach for semantic segmentation tasks.

Mohan et al. (2022) [145] proposed an enhanced pothole detection system based on the YOLOX algorithm, marking one of the first applications of the YOLOX object detection method for pothole detection. The authors introduced the latest YOLOX object detection algorithm, optimizing model performance via anchor point elimination, data augmentation, and SimOTA label matching strategies. The experiment utilized a dataset of 665 labeled images, which were preprocessed for training the YOLOX-Nano model. Results showed that its average precision (AP) reached 85.6%, with the model size being only 7.22 MB, outperforming lightweight models such as YOLOv5s and YOLOv4-tiny. Although its inference speed (0.038 s) was slightly lower than that of some comparison models, its high accuracy and small size render it suitable for real-time mobile deployment.

Ugenti et al. (2022) [146] constructed an efficient feature subset via physics-driven signal enhancement and feature selection algorithms, which was used to train a SVM classifier. This approach was compared with a CNN based on signal spectrograms. Experiments were validated using the SherpaTT rover across diverse terrains, including sandy, gravel, and paved surfaces. The results demonstrated that both methods achieved an accuracy exceeding 90% in generalization tasks; however, the CNN significantly outperformed the SVM in extrapolation tasks (e.g., varying speeds or unknown terrains). This study highlights the importance of feature selection in reducing computational costs while preserving classification performance, provides a technical foundation for long-term autonomous navigation in future planetary exploration missions, and may further serve as a reference for autonomous driving technologies.

Kumari et al. (2023) [147] pre-trained YOLOv8 on the large-scale COCO dataset, then fine-tuned it on the pothole dataset to evaluate YOLOv8’s performance. Different YOLOv8 variants were used to analyze and compare results on the dataset, with various datasets of pothole images captured under diverse weather conditions added to enhance model performance. The dataset was split into training, validation, and test sets at a 70:15:15 ratio, comprising 5600 training images, 1200 validation images, and 1200 test images. Experimental results showed that YOLOv8x and YOLOv8l performed better, with precision of 83.2% and 82.6%, respectively.

Aishwarya et al. (2023) [148] proposed a visual speed bump detection method based on Faster R-CNN and YOLOv5, focusing on detecting both labeled and unlabeled speed bumps in real environments. To address the issue of misclassifying pedestrian crossings as speed bumps, a negative sample training (NST) strategy was introduced to optimize the model, improving speed bump detection accuracy. Experiments showed that NST increased the average accuracy of YOLOv5 for labeled speed bumps by 2.3%, with the final model achieving an mAP@0.5 of 98.8%. The inference speed on the NVIDIA Jetson platform reached 18.5 FPS, enabling effective distinction between speed bumps and crosswalk lines. The YOLOv5+NST model, with a size of only 42.2 MB, is easy to integrate into vehicles for real-time deployment. To verify its effectiveness, the model was tested in a real campus environment.

Heo et al. (2023) [149] proposed a real-time pothole detection method based on a multi-scale feature network (SPFPN-YOLOv4-tiny). This method integrates spatial pyramid pooling (SPP) and feature pyramid network (FPN) into the CSPDarknet53-tiny backbone network of YOLOv4-tiny to enhance multi-scale feature extraction capability. K-means++ clustering is used to optimize anchor box sizes, improving detection accuracy. Finally, by combining a monocular camera and distance estimation algorithm, the 2D size of detected potholes is converted into actual 3D sizes. Experiments show that SPFPN-YOLOv4-tiny improves average precision (mAP@0.5) by 2–5% compared to YOLOv2, YOLOv3, and YOLOv4-tiny, reaching 79.6%. Real-time detection speed in a CPU environment reaches 38 FPS.

Hussein et al. (2024) [150] proposed an intelligent speed bump detection method based on the YOLOv8 algorithm. The research team collected real road data to construct an 8973-image dataset (including speed bumps with/without markings), dividing it into training (77%), validation (20%), and testing (3%) subsets. Experiments showed that the YOLOv8n model achieved the highest detection accuracy on the Jetson Nano edge device for both marked and unmarked speed bumps, with an average precision (mAP@0.5) score of 0.81. Notably, this method integrated a Kinect Xbox camera with the Jetson Nano developer kit to enable distance estimation for detected speed bumps.

Dewangan et al. (2024) [151] proposed a speed bump detection network (SBDNet) based on regional proposal and non-maximum suppression to accurately identify speed bumps while addressing misclassification issues between speed bumps and zebra crossings in visual-based driving assistance systems. The study utilized 543 images from the public “Speed Bump/Speed Bump Dataset” and expanded it to 3258 images through data augmentation techniques. Experimental results showed that SBDNet achieved an accuracy of 99.34%, sensitivity of 99.52%, and F1-score of 99.04%. Compared with state-of-the-art methods such as YOLOv8 and SSD, SBDNet demonstrated superior performance in detecting both marked and unmarked speed bumps, with the highest detection accuracy among evaluated CNNs.

Bodake and Meeeshala (2025) [152] proposed FTayCO-DCN, an integrated framework combining the Crocuta algorithm, fractional Taylor optimization, and deep convolutional neural networks (DCN) for road pothole detection. It uses FTayCO to optimize DCN hyperparameters and integrates aerial image transformation, ROI extraction, and Otsu threshold preprocessing to enhance feature extraction. On a 320 image dataset (240 training, 40 validation, 40 testing), it achieved 99.06% accuracy, 99.09% sensitivity, and 99.04% specificity, outperforming YOLOv8 and SSD by 2–3% in accuracy. Notably, it reached 98.2% mAP@0.5 for small potholes (≤30 cm in diameter) at over 5 m, addressing long-range detection gaps.

Chandak et al. (2024) [153] utilized, trained, and optimized SSD and YOLO architectures on diverse datasets, employing data augmentation to enhance pothole detection accuracy. They collected extensive datasets, trained the models, and evaluated metrics including accuracy, recall, and F1-score. Experiments showed that YOLOv4 achieved a relatively high mAP@0.5 of 85.48%. In contrast, SSD-MobileNet achieved an accuracy of 0.42, recall of 0.81, and F1-score of 0.82, showing distinct advantages in certain metrics. However, in terms of processing speed, SSD-MobileNet (7 ms) outperformed YOLOv4 (52 ms). Combining these two models can thus yield a more reliable and efficient detection system, suitable for applications requiring timely and accurate object recognition—such as autonomous vehicles, drones, and other safety-critical systems. Table 6 summarizes research on object detection using machine learning and deep learning methods.

Currently, in the field of road surface information detection, machine learning and deep learning technologies are widely adopted, with core methods centering on efficient target detection frameworks. Mainstream frameworks include single-stage models (e.g., the YOLO series, SSD) and two-stage models (e.g., Faster R-CNN [136], Mask R-CNN [154]). Through training, these frameworks can detect and localize potholes and speed bumps, significantly enhancing perceptual robustness in complex environments. The core advantage of these methods lies in pre-acquiring geometric features of the upcoming road via visual sensors, overcoming the lag-related limitations of traditional dynamic methods, and creating a millisecond-level control window for suspension preview. Additionally, lightweight models (e.g., YOLOX-Nano [53], Tiny-YOLOv4 [155]) are deployed on edge computing platforms, maintaining over 90% detection accuracy while meeting real-time requirements (>30 FPS) to support preview decisions under high-speed conditions. However, their performance remains limited by the distribution of training data, tending to produce false detections under extreme lighting conditions.

### 3.5. Multi-Sensor Fusion Methods

Multi-sensor fusion can realize the complementary advantages of different types of data information so as to solve the problem of insufficient perception accuracy caused by the limitation of a single-modal data. At present, 2D image data and 3D point cloud data captured by cameras and lidar separately are commonly used in fusion algorithms. The camera data are rich in texture, color, and category-discriminative information of the target, and the point cloud data can provide 3D information, such as the contour, distance, and position of the target in the perception environment. In fusion models, branches of different modalities independently perform the same or different degrees of feature encoding, and then the generated data are fused with certain strategies. The input of the fusion process is known to have various types, such as raw data, features, and results, which are correspondingly produced at different stages of feature encoding branches. The level of data of each modality in the fusion process actually determines specifically used fusion strategies. For example, integrating 2D image processing with 3D point clouds not only reduces computational resource consumption but also facilitates the acquisition of 3D contour information, thus achieving efficient road surface information detection [156]. With the advancement of deep learning, models like YOLO [157] and Mask R-CNN can detect the area and depth of road potholes, facilitating the integration of different methods to form more robust models [158].

Joubert et al. (2011) [156] designed a low-cost onboard automatic road pothole detection system. This system integrates 3D point cloud data collected by Microsoft Kinect sensors with 2D images acquired by high-speed USB cameras, and incorporates GPS positioning to analyze geometric features and mark pothole positions. Using RANSAC plane estimation to separate the road surface from pothole point clouds and integrating image edge detection to calculate pothole width and depth, the system achieves a measurement error of less than 0.5 cm.

Li et al. (2016) [159] proposed an automated pothole detection method based on the fusion of images and ground-penetrating radar (GPR) data. First, a data acquisition system was constructed using RTK GPS, high-definition cameras, and GPR. The collected GPR data was denoised, while the images were smoothed and background light was normalized. Next, a three-level filter based on GPR signal characteristics was designed to achieve preliminary pothole detection. Finally, the pothole shape was extracted from the images using the geometric active contour model (Chan-Vese algorithm) and morphological operations, combined with GPR positioning information. Verified on 50 datasets, the method achieved 94.7% precision, 90% recall, and 88% accuracy, with an average shape extraction error of 12.8%. This study also indicated that the method exhibits better performance in complex backgrounds.

Kang and Choi (2017) [160] proposed a detection method based on the combination of 2D radar and cameras. The authors adopted a heterogeneous sensor fusion strategy: 2D LiDAR obtained the width and depth of potholes through noise filtering, clustering, line extraction, and data gradient analysis. The color camera extracted pothole shape information through Gaussian filtering, brightness adjustment, binarization, and Canny edge detection. Experiments showed that the dual-LiDAR layout effectively reduced the detection error rate (<5%) and achieved 3D pothole reconstruction in moving scenarios. After fusing data from the two sensor types (2D LiDAR and cameras), the system exhibited higher robustness under complex road conditions.

Yun et al. (2019) [161] proposed a two-level ramp detection method. The first level uses a Haar cascade classifier to extract candidate regions from binary images; the second level verifies these regions by fusing LiDAR point cloud HOG features with an SVM classifier, while employing coordinate transformation to align sensor data. Experiments demonstrated that this method achieved an average detection rate of 85.2% across multiple scenarios in South Korea, with the false alarm rate reduced by approximately 30% compared to single-detector methods. Its advantages include enhanced classification robustness through candidate region screening and the ability to obtain ramp height information via LiDAR. However, the processing time increased to 30 ms (10 ms longer than single-detector methods) and the method is sensitive to worn ramp patterns.

Salaudeen and Celebi (2022) [162] proposed a pothole detection method integrating the Enhanced Super-Resolution Generative Adversarial Network (ESRGAN) with object detection models to address the insufficient performance of small object detection in low-resolution images. First, ESRGAN is used to conduct 4× super-resolution reconstruction on low-resolution road images, thereby enhancing texture details and edge information. Subsequently, two object detection models, namely YOLOv5 and EfficientDet, are leveraged for training and inference on the enhanced high-resolution images. The experiment utilized 1300 images from the CCSAD, PNW, Japan Pothole, and Sunny datasets. The effectiveness of the method was validated by comparing the detection performance of super-resolution (SR) and low-resolution (LR) images. The results demonstrated that the performance of models trained on SR images was significantly improved: YOLOv5 achieved 97.6% in mean Average Precision (mAP@0.5) and 70% in recall rate, marking an approximate 30% improvement compared to those trained on LR images. Furthermore, the method exhibited superior performance in scenarios with complex lighting conditions and the distant small potholes.

Roman-Garay (2025) [163] proposed a pavement pothole assessment framework integrating deep learning, machine vision, and fuzzy logic. Intel RealSense D435i cameras were used to collect 2D images and 3D point clouds, with a dataset constructed containing 583 images (including environmental noise) labeled via semantic segmentation, where each image corresponds to its respective point cloud. The model leveraged transfer learning with the Segformer network to enhance detection performance, achieving a high intersection-over-union (IoU) score that enables accurate diameter estimation of potholes. Experiments using road data demonstrated a recall of 90.87%, accuracy of 90.01%, F1-score of 90.43%, and a loss value of 0.0431. Additionally, the framework integrated image data and point cloud data to estimate pothole size and depth, with a depth error of 5.94 mm. Table 7 summarizes studies employing multi-sensor fusion methods.

Multi-sensor fusion methods fusing 2D and 3D enable rapid identification and localization of road potholes and speed bumps via 2D technology, while precisely quantifying geometric parameters (e.g., depth, height) using 3D technology. This approach compensates for the lighting invariance limitations of 2D vision through 3D modeling and combines complementary information from 2D texture semantics and 3D geometric structures, significantly enhancing the comprehensiveness and accuracy of perception. However, challenges remain in the robustness of spatiotemporal synchronization for heterogeneous data and cross-modal feature alignment.

### 3.6. Comparative Analysis and Challenges

While various detection technologies for pavement information perception have been discussed, each presents distinct advantages and challenges. For example, traditional dynamic methods are cost-effective and easy to deploy but fail to meet real-time requirements. Two-dimensional image processing features processing simplicity and low cost, but is susceptible to poor lighting conditions and complex road conditions, and lacks 3D contour information. The 3D point cloud method provides 3D contour data but requires substantial time for point cloud processing and reconstruction. Machine learning/deep learning, despite demonstrating high accuracy, reliability, and robustness, relies heavily on large-scale training data. Multi-sensor fusion methods, while addressing the limitations of single sensors and achieving high accuracy, introduce integration complexity. Table 8 summarizes the advantages and disadvantages of each method.

Based on findings from the reviewed literature, Table 9 presents a comparative analysis of the average precision, recall, F1 score, and computational cost for each detection method under study. The multi-sensor fusion method has exhibited well-rounded and high-performance detection indicators. However, such high-performance indicators are achieved at the cost of increased computational complexity and heightened system integration difficulty. By contrast, deep learning techniques strike a balance between performance and efficiency, thus emerging as the optimal candidate for real-time detection applications.

In practical pavement detection scenarios, these technologies also confront prominent environmental challenges that directly impair their application performance, particularly under extreme weather conditions. For traditional dynamic methods, strong winds can induce vibrations in vehicle-mounted detection equipment, resulting in unstable data acquisition and distorted pavement condition records; frost accumulation on the equipment’s contact components may further increase frictional resistance, diminishing the responsiveness of dynamic detection systems. For 2D image processing systems, rainwater splashing on camera lenses creates water film interference, snowflakes adhering to the lenses block the field of view, and frost formation leads to blurred image edges—all of which hinder the accurate identification of fine pavement cracks or potholes. The 3D point cloud method is not immune either: heavy rain can scatter laser signals emitted by sensors, causing sparsity or gaps in point cloud data for local pavement areas, while strong winds may slightly shift the sensors’ mounting positions, inducing deviations in the 3D contour reconstruction of pavement surfaces. For machine learning/deep learning models, the scarcity of labeled pavement data under extreme weather such as snow-covered or rain-wet pavements impedes the model’s learning of representative features of abnormal pavement conditions in such scenarios, leading to a significant decline in detection accuracy when deployed in real-world harsh conditions. Even multi-sensor fusion methods encounter obstacles: disparities in the environmental adaptability of various sensors exist, for instance, optical sensors are sensitive to rain and snow, whereas inertial sensors are susceptible to wind-induced vibrations. These disparities elevate the difficulty of data calibration and fusion, and may trigger conflicts in fused results, reducing the overall reliability of pavement detection.

To mitigate these practical challenges, targeted optimization strategies can be implemented for different technologies. For traditional dynamic methods, installing shock-absorbing brackets on vehicle-mounted equipment to mitigate wind-induced vibrations and integrating low-temperature-resistant heating elements into contact components to prevent frost accumulation can enhance the stability of data acquisition. For 2D image processing systems, coating camera lenses with hydrophobic and anti-frost films to minimize water film and frost interference, and incorporating real-time image enhancement algorithms including adaptive histogram equalization to recover details of images blurred by snow or rain help improve detection accuracy. For the 3D point cloud method, using high-power anti-interference laser sensors to reduce signal scattering in rainy conditions and incorporating real-time attitude correction algorithms based on inertial measurement units (IMUs) to compensate for position deviations induced by strong winds can optimize the quality of point cloud data. For machine learning/deep learning models, adopting data augmentation techniques such as simulating snow coverage or rain streaks to augment extreme weather datasets and introducing transfer learning to migrate knowledge from normal-weather models to extreme scenarios can alleviate the scarcity of labeled data and enhance model generalization. For multi-sensor fusion methods, designing adaptive weight assignment mechanisms that modulate the contribution of each sensor based on real-time environmental conditions, for example, the weight of optical sensor data can be reduced in heavy rain, and developing multi-modal data calibration algorithms compatible with extreme weather conditions can resolve data conflicts and improve fusion reliability.

## 4. Elevation Information Detection

Suspension preview control, as a core technology of the intelligent chassis system, relies heavily on the accuracy of forward road information perception [27,164]. To address this limitation, the suspension preview system pre-acquires elevation contour information of the upcoming road (e.g., pothole depth and speed bump height), enabling forward-looking regulation of the suspension system. Using high-precision depth information, the system can calculate vehicle posture changes and generate active control strategies, thereby enhancing vehicle ride comfort and handling stability when traversing uneven roads [165]. Thus, the perception and modeling of road elevation information are core prerequisites for performance breakthroughs in preview suspension systems.

Current research in this field includes the following studies: Lee (2018) [166] used LiDAR and IMU sensors to detect speed bumps, applying this information to semi-active control of the front-axle suspension system in agricultural tractors. Seok (2024) [167] employed adaptive smoothing and curvature analysis on LiDAR point cloud data to detect road bumps, aiming to enhance preview suspension performance. By introducing ground point cloud extraction technology, the method effectively distinguishes drivable areas from non-ground obstacles, with a height estimation error of less than 6 mm. Additionally, Kang (2024) [168] proposed a method using a bidirectional LSTM model and IMU data, combined with cloud-based continuous learning, to predict remaining distance and bump height—achieving a predicted distance root mean square error (RMSE) of 6.37 m and a height error of 0.9 cm. This approach provides a new direction for low-cost, robust suspension preview systems.

Currently, machine vision-based vehicle suspension preview control is in its infancy. Few studies have integrated road elevation information detection with suspension preview control systems; most focus on road pothole depth detection, with less attention to speed bump height detection. Thus, the following section summarizes the relevant literature on road elevation information to serve as a reference.

Chitale et al. (2020) [169] proposed a pothole detection and size estimation system based on the YOLOv4 algorithm and triangular similarity image processing. This system adopts a two-stage approach: First, it uses the YOLOv4 model for pothole detection, enhancing IoU performance via the introduction of CIoU and DIoU loss functions to achieve an mAP of 0.933, 6% higher than YOLOv3. Subsequently, leveraging the camera height (90 cm) and triangular similarity principle, it estimates the actual size of potholes based on pixel ratios, with an average error rate of 5.868%. Experiments utilized a custom dataset containing 1300 multi-scenario images, covering complex conditions (e.g., water stains, dry surfaces) and validating the method’s robustness.

Ahmed (2021) [170] proposed a low-cost 3D reconstruction method for road pot-holes based on structure from motion (SFM) and laser triangulation measurement. To address scale ambiguity in traditional monocular reconstruction, the method innovatively combines a monocular camera with a laser indicator: it generates 3D point clouds using SIFT feature matching and the 5-point algorithm, and resolves scale ambiguity via the laser indicator and triangulation measurement. Experimental results show that under static imaging, average pothole depth and perimeter errors are 5.3% and 5.2%, respectively; in dynamic tests (vehicle speeds: 10–20 km/h), depth error in-creases to 26.6% and perimeter error reaches 27.8% as speed rises. Verified using artificial depression benchmarks, the method achieves an average depth error of 3.0% and perimeter error of 7.7%.

Das and Kale (2021) [171] proposed a novel pothole detection algorithm P3De based on 3D depth estimation, aiming to enhance real-time detection accuracy and practicality through dynamic road depth analysis. The algorithm combines a convolutional neural network (CNN) and a region proposal network (RPN), extracts video features, and generates candidate regions. It uses gradient changes to distinguish potholes from cracks. Via frequency heatmap calibration technology, it converts real-time videos into VIBGYOR color-level heatmaps, mapping road depth differences via colors to enhance the system’s adaptability to complex environments. Experiments, based on the KITTI stereo dataset, optimize the model training process and use the cross-entropy loss function and Adam optimizer to enhance performance.

Li et al. (2022) [172] proposed a 3D pavement reconstruction method integrating genetic algorithm-optimized DenseNet (GA-DenseNet) with binocular stereo vision. By combining binocular stereo vision technology, the method uses point cloud interpolation and plane fitting algorithms to extract 3D pothole features. The average accuracy of depth and area detection is 98.9% and 98%, respectively. For the first time, it calculates the pavement damage rate (DR) and pavement condition index (PCI) from RGB images with millimeter-level accuracy, achieving an overall detection accuracy of 88.2%.

Wang (2023) [78] proposed a 3D pavement pothole reconstruction and segmentation system based on improved structure from motion (SFM) and deep learning. The PP-SFM method is proposed, which uses multi-view 2D images to generate dense point cloud data, enabling low-cost, high-precision 3D reconstruction. For point cloud segmentation, the Trans-3DSeg network is designed, introducing a Transformer module to enhance global feature perception and fusing low-order and high-order features to improve segmentation accuracy. Experiments show that the improved system achieves 93.44% accuracy and a 92.58% F1 score on the test set, approximately 2.41–4.13% higher than methods such as PointNet++ and PointRCNN, and exhibits good robustness under low-light conditions and varying shooting heights.

Ranyal et al. (2023) [173] improved the single-stage CNN RetinanNet and proposed an intelligent 3D visual pavement pothole detection system. This system extracts frames from pavement videos, employs structured photogrammetry to simulate pothole 3D point clouds for depth evaluation, and integrates with the CNN-based pothole detector. It successfully detects potholes, achieving an F1 score of up to 98% on the dataset, with average pothole depth estimation error below 5%.

Singh et al. (2024) [174] introduced a real-time model for pothole detection and depth estimation using computer vision and deep learning. The main approach uses the Faster R-CNN ResNet-50 FPN model for real-time pothole detection, with training and validation accuracies of 96% and 85%, respectively. Additionally, via image processing techniques, the algorithm leverages a smartphone camera and an ultrasonic sensor to identify potholes and estimate their depths. The MIDAS model accurately calculates distances between the pothole and its surrounding environment, thereby estimating pothole depth. It identifies potholes in diverse environments and overcomes limitations of existing systems.

Park and Nguyen (2025) [175] proposed a real-time pavement pothole detection method based on dual-camera vision technology and the YOLOv8 instance segmentation model (YOLOv8-seg). By combining a dual-camera system with optical geometry principles, the method calculates real-time pothole area and depth, and employs laser-assisted measurement to enhance depth detection accuracy. Experiments show it has an error rate below 5% and excels in contour recognition of complex-shaped potholes. Moreover, it enables real-time detection and distance measurement, as well as size and shape evaluation of target objects. Thus, this technology is highly suitable for applications such as autonomous driving systems, advanced driver assistance systems (ADAS), and other vehicle safety technologies. Table 10 summarizes studies providing elevation information.

Accurate elevation information, specifically the depth of potholes and the height of speed bumps, is paramount for effective suspension preview control systems. This section synthesizes research focused on acquiring this critical 3D contour data. Reviewed techniques include LiDAR point cloud analysis, stereo vision reconstruction, combining object detection models such as YOLO with triangulation or stereo imaging for size/depth estimation, SFM with laser scaling, and dedicated depth estimation algorithms using CNNs and disparity maps. These studies demonstrate various approaches for quantifying depression depth or protrusion height. While progress in pothole depth detection is evident, research specifically targeting speed bump height estimation remains relatively scarce. Providing precise elevation data enables the suspension system to proactively compute necessary adjustments, enhancing ride comfort and stability over uneven road surfaces.

## 5. Future Outlook

Although computer vision and deep learning have made significant progress in pavement information detection, further research still faces challenges including limited robustness in multi-modal fusion, weak generalization ability in extreme scenarios, and low computational efficiency. Specifically, heterogeneous sensors (e.g., cameras, LiDAR, and millimeter-wave radars) tend to cause temporal-spatial synchronization errors due to large differences in sampling frequency and coordinate systems, reducing the accuracy of pavement elevation modeling. Notably, in extreme conditions such as heavy rain, heavy snow, or strong glare, existing models show significant performance degradation, with false detection rates increasing by orders of magnitude. Moreover, the high computational complexity of high-precision point cloud reconstruction and semantic segmentation forces the system to balance detection accuracy and processing latency, making it difficult to meet the strict millisecond-level response requirements of suspension preview control. Breaking through these technical bottlenecks is crucial for improving the accuracy, robustness, and real-time performance of pavement perception, thereby optimizing vehicle preview control, enhancing ride comfort, and improving handling stability.

Future research should focus on developing more robust, efficient, and scalable pavement perception systems. Key directions include: (1) Deeply integrating multi-source heterogeneous sensors (e.g., millimeter-wave radars, thermal imaging cameras, IMUs, and wheel speed sensors) to break through cross-modal feature alignment and adaptive fusion mechanisms, leveraging the harsh weather penetration capability of millimeter-wave radars and the temperature sensitivity of thermal imaging to achieve all-weather, full-scenario perception. (2) Continuously advancing model lightweighting and efficient deployment through hardware-customized neural architecture search (NAS), knowledge distillation (KD), model pruning/quantization, and operator-level optimization to meet the strict requirements of on-vehicle platforms for real-time performance and power consumption. (3) Emphasizing the development of weakly supervised and self-supervised learning strategies (such as semi-supervised learning, domain adaptation, and few-shot learning) to effectively utilize simulated and unlabeled data, thereby significantly reducing reliance on fine-grained annotations. (4) Deepening collaborative perception enabled by vehicle–road collaboration, integrating on-vehicle and roadside perception devices to expand spatiotemporal perception boundaries and improve perception reliability and accuracy. Meanwhile, it addressing key technologies including efficient and reliable data transmission, real-time fusion of heterogeneous data, and information security and privacy protection. (5) Proprioceptive sensing is defined as the perceptual capacity to acquire physical signals pertaining to its own motion states and the interactions between its limbs (or actuators) and the environment via onboard sensors. This sensing paradigm can be leveraged to tackle core detection tasks such as force measurement and vibration sensing. Notably, learning algorithms trained directly on raw proprioceptive signals or their derived feature sets enable the inference of terrain properties and the prediction of observations under previously unseen conditions (e.g., novel traversal speeds or terrain types). Furthermore, a feature selection algorithm based on a scoring system and iterative search facilitates the preservation of high classification accuracy while mitigating computational burdens [173]. (6) Most existing studies operate under the assumption that sensor data are acquired reliably and of high quality, whereas real-world scenarios are plagued by data loss and compromised data quality. In practice, key information is often occluded by vehicles and buildings; data quality degrades with increasing distance and is significantly impaired by extreme weather conditions; and the high-speed movement of objects further complicates the spatiotemporal alignment of sensory data. A single type of sensor is ill-equipped to cope with dynamic and harsh environmental conditions, whereas multi-sensor fusion can not only enrich the pool of available environmental information but also enhance the robustness of algorithmic systems against the challenges posed by weather, distance, and poor data quality. However, naive data fusion strategies are still not sufficient for applications. In practical scenarios, different vehicles may be equipped with various numbers and types of sensors with different sensor characteristics and data quality, which leads to serious long tail phenomena. Hence, a generic cooperative perception framework with variable modal types and numbers of traffic participants and robust fusion strategies needs to be thoroughly explored in future work. Breakthroughs in these directions will significantly enhance the performance and practicality of pavement information perception systems.

In summary, the future development of pavement information detection technology is a process of continuous innovation that integrates interdisciplinary frontiers and addresses practical engineering challenges. Breakthroughs are required in multiple dimensions, such as deep integration of perception, enhanced computing efficiency, innovative data utilization, improved scene robustness, precise elevation reconstruction, and system-level collaboration. Only then can the core value of the intelligent suspension preview system be truly realized. This will further drive a qualitative leap in ride comfort, vehicle safety, and energy economy.

## 6. Conclusions

This paper begins by elaborating on the importance of pavement perception for enabling the predictiveness and active adjustability of the suspension preview system, highlighting the engineering value and research prospects of the study. At the dataset level, we have sorted out various sensors supporting the development of this research and summarized mainstream public dataset resources, providing a crucial data foundation for algorithm development and performance evaluation. In the section on core detection methods, we outline five approaches: traditional dynamics, 2D image processing, 3D point cloud analysis, machine learning and deep learning, and multi-sensor fusion methods. Each method has its own advantages and disadvantages, but collectively they offer multifaceted strategies for pavement information detection. Methods based on traditional vehicle dynamics response analysis employ physical signals generated by vehicle–road interaction for indirect inference. Their strength lies in not relying on external sensors, yet they are susceptible to interference from driving behavior and loads. Two-dimensional vision-based methods, leveraging mature image processing, feature high cost-effectiveness and rich information, though the lack of depth information limits their direct application in preview control. Three-dimensional point cloud-based methods can directly acquire spatial geometric information of targets with high precision, but are constrained by cost, weather influences, and computational complexity. Machine learning and deep learning-based methods demonstrate strong feature learning capabilities, with significant potential in complex scene understanding, making them a current key research hotspot. Multi-sensor fusion methods aim to integrate the strengths of the aforementioned approaches, enhancing the overall system’s robustness and adaptability through information complementarity, representing a more practically valuable development direction. Furthermore, this paper specifically discusses elevation information detection, a core technology directly related to suspension preview, emphasizing the irreplaceability of providing accurate elevation information for preview control algorithms to generate optimal control commands.

Finally, a summary and outlook of road surface information detection methods are presented. For application scenarios of suspension preview that demand high real-time performance and high reliability, several future research directions are outlined: (1) fusion of multi-modal data; (2) research on lightweight models; (3) development of weakly supervised and self-supervised learning strategies; (4) expansion of perception capabilities enabled by vehicle–road collaboration; (5) proprioceptive sensing application; (6) generic cooperative perception framework. Road surface information perception technology must achieve breakthroughs in key directions. Only then can it mature and be deeply integrated into next-generation intelligent suspension preview systems. Ultimately, this technology will provide solid technical support for enhancing vehicle ride comfort, ensuring driving safety, and extending the service life of chassis components.

## Figures and Tables

**Table 1:** Open-access Dataset for Pothole Detection.

Dataset Name	Description	Train	Validation	Test	Access Link
The Pothole-600 Dataset	Provide disparity maps converted using stereo matching algorithms	239	179	179	Google (https://sites.google.com/view/pothole-600/dataset; accessed on 1 September 2025)
Dib’s Pothole Detection Dataset	The first dataset with water-filled and dried pothole	570	173	/	Mendeley (https://data.mendeley.com/datasets/tp95cdvgm8; accessed on 1 September 2025)
PODS Dataset	Including potholes in various road environments	1191	113	59	Universe (https://universe.roboflow.com/pods/pothole-detection-x7dgh; accessed on 1 September 2025)
Abhinva Kulshreshth’s Pothole Detection Dataset	Contains both normal images and potholes images, combines of Google and Kaggle	1167	108	136	Kaggle (https://www.kaggle.com/datasets/abhinavkulshreshth/pothole-detection-dataset; accessed on 1 September 2025)
Semantic Segmentation of Pothole and Cracks Dataset	A dataset focused on semantic segmentation	3340	496	504	DeepLearning (http://deeplearning.ge.imati.cnr.it/genova-5G/; accessed on 1 September 2025)
Japanese Road Damage Detection Dataset	Images of road damage instances captured using a mobile phone installed in the vehicle	7718	4630	3087	GitHub (https://github.com/sekilab/RoadDamageDetector; accessed on 1 September 2025)

**Table 2:** Public Dataset for Speed Bump Detection.

Dataset Name	Description	Total Images	Access Link
SpeedHump/bumpDataset	Images of marked and unmarked speed bumps	543	Mendeley (https://data.mendeley.com/datasets/xt5bjdhy5g/1; accessed on 1 September 2025)
Marked Speed Bump/Speed Breaker Dataset (India)	Image of speed bumps on Indian roads	969	Mendeley (https://data.mendeley.com/datasets/bvpt9xdjz8/1; accessed on 1 September 2025)
ZIYA’s Speed Bump Detection Dataset	Including images of speed bumps, potholes, cracks and normal roads under various road conditions	270	Kaggle (https://www.kaggle.com/datasets/ziya07/speed-bump-dataset/data; accessed on 1 September 2025)
Universe Speed Bump Dataset (4)	Contains the images used for the object detection of the YOLO model	593	Universe (https://universe.roboflow.com/alia-khalifa/detecting-speed-bumps/dataset/4; accessed on 1 September 2025)
Universe Speed Bump Dataset (15)	One of the largest datasets for speed bumps object detection	1692	Universe (https://universe.roboflow.com/speed-bump-detection/speed-bump-detection-se0eh/dataset/15; accessed on 1 September 2025)

**Table 3:** Review of Studies using Traditional Dynamic Methods.

Authors	Date	Method	Descriptions	Performance
Mednis et al. (2011) [86]	Acceleration signal	Z-THRESH, Z-DIFF, STDEV(Z), G-ZERO	Four algorithms were used to analyze acceleration signals. Among them, the Z-DIFF algorithm achieved the best performance.	Positive rate: 99%
Wang et al. (2015) [87]	Acceleration signal, GPS	Normalization, Z-THRESH Spatial Interpolation Method, G-ZERO	Acceleration data were normalized to establish a reference angle, and the Z-THRESH and G-ZERO algorithms were integrated to enhance detection accuracy.	False positive: 0%
Rishiwal and Khan (2016) [88]	Acceleration signal	*Z*-axis acceleration threshold	By detecting the sudden change in *Z*-axis acceleration, combined with threshold detection and classification.	Accuracy: 93.75%
Aljaafreh et al. (2017) [89]	Acceleration signal	Fuzzy Inference System (FIS)	The fuzzy reasoning system detects speed bumps via vehicle vertical acceleration and speed changes.	/
Celaya-Padilla et al. (2018) [91]	Acceleration signal, gyroscope data, GPS	Genetic algorithm, cross-validation	Retrieving data from sensors, employing a cross-validation strategy, and using the genetic algorithm for speed bump detection.	Accuracy: 97.14%
Rodrigues et al. (2019) [92]	Acceleration signal	Haar wavelet transform (HWT), two-step thresholding procedure, adaptive threshold estimation	Wavelet coefficients are obtained via a two-step thresholding process, with adaptive threshold estimation employed instead of manual threshold calibration.	/
Lekshmipathy et al. (2021) [93]	Acceleration signal	High-pass filtering, algorithm combination	By determining the optimal combination and thresholds of different algorithms, the proposed algorithm-threshold combination achieved a true positive rate of 93.18%.	True positive: 93.18% False positive: 20%
Yin et al. (2024) [96]	Acceleration signal, GPS	Feature extraction filter (EFDD), least squares method, Euler point, wavelet technology, genetic algorithm	A new method for detecting speed bumps using accelerometers, GPS sensors, and feature extraction filters (EFDD) achieves 100% accuracy in speed bump detection.	Pothole accuracy: 75% Speed bumps accuracy: 75%
Zhang et al. (2025) [97]	Acceleration signal	Newmark method, particle swarm optimization algorithm	The acceleration when passing through potholes is derived by solving the vibration equation via the Newmark method, and pothole depth is inversely estimated using the particle swarm optimization algorithm.	Average error rate: 8.94%

**Table 4:** Review of studies employing 2D methods.

Authors	Date	Method	Descriptions	Performance
Buza et al. (2013) [105]	Color image	Otsu’s threshold processing, spectral clustering algorithm	By using the data based on the histogram in the grayscale image, spectral clustering is employed to identify the potholes.	Accuracy: 81%
Kiran and Murali (2014) [106]	Color image	Canny edge detection, Hough transform, morphological processing	Speed bumps are detected using Canny edge detection, Hough transform, and morphological processing.	/
Ryu et al. (2015) [107]	Color image	Based on histogram, thresholding, geometric features	Using histograms and morphological closing operations, dark areas for pothole detection are extracted; pothole contours are extracted using geometric features.	Accuracy: 71.6% Precision: 85.3% Recall: 61.3%
Devapriya et al. (2015) [108]	Color image	Grayscale conversion, binarization, morphological processing, horizontal projection method	Speed bumps are detected using grayscale conversion, binarization, morphological processing, and the horizontal projection method.	Ture positive: 92%
Schiopu et al. (2016) [109]	Color image	Histogram-based threshold, geometric properties	Select the region of interest (ROI) and employ a threshold-based algorithm to generate it.	Precision: 90% Recall: 100%
Devapriya et al. (2016) [110]	Color image	Gaussian filtering, median filtering, connected region analysis	Methods using Gaussian filtering, median filtering, and connected region analysis to detect speed bumps yield relatively low detection accuracy for unmarked ones.	Accuracy: 85%
Ouma and Habn (2017) [111]	Color image	Wavelet transform, fuzzy c-means clustering, morphological reconstruction	Using wavelet transform to reduce noise, fuzzy C-means clustering extracts pothole areas, and morphological reconstruction optimizes pothole edge detection.	Accuracy: 87.5%
Srimongkon and Chiracharit (2017) [112]	Color image	Gaussian mixture model, morphological processing	Based on Gaussian mixture model segmentation and morphological operations, speed bumps are identified and detected.	Ture positive: 82.75% False positive: 17.25%
Wang et al. (2017) [113]	Grayscale Image	Wavelet energy field, Markov random field, morphological processing	Construct the wavelet energy field of road surface images, detect potholes using morphological processing, and perform segmentation using the Markov random fields.	Precision: 85.7% Recall: 72% F1: 78.3%
Sirbu et al. (2021) [115]	Color image	Gaussian filtering, semantic segmentation, ED line algorithm	Apply Gaussian filtering to smooth the image, extract the region of interest based on semantic segmentation, and use the ED algorithm for speed bump detection.	Accuracy: 71.6% Precision: 85.3% Recall: 61.3%

**Table 5:** Review of studies employing 3D methods.

Authors	Date	Method	Descriptions	Performance
Fernández et al. (2012) [122]	3D road point cloud	Coordinate system conversion, free space detection algorithm	LiDAR provides four horizontal layer measurements, combined with the free space detection algorithm to detect speed bumps.	Computed time: 6.48 ms
Moazzam et al. (2013) [123]	Depth image	Coordinate system transformation, trigonometric surveying method	The Kinect is used to capture depth images, enabling low-cost acquisition of pit depth without complex calculations.	Low cost
Melo et al. (2018) [124]	Radar scanning data	Interferometric measurement method, SFCW	Innovative integration of radar interferometry with SFCW enables effective speed bump detection and height measurement.	height estimation error less than 5%
Tsai and Chatterjee (2018) [125]	3D road surface data	Data correction, watershed algorithm	The collected 3D road data are corrected, with potholes detected via the watershed algorithm.	Accuracy: 94.97% Precision: 90.80% Recall: 98.75%
Lion et al. (2018) [126]	Color images, depth images	Three-dimensional scene reconstruction, morphological processing, Canny edge detection	Using the Kinect to obtain ground color and depth images enables three-dimensional scene reconstruction, enabling effective detection of speed bumps’ height and distance.	Accuracy: 86.84%
Wu et al. (2021) [127]	3D road point cloud	GPT-SGM, Three-Stage Normal Filter (3F2N), Discriminative Scale Space Tracking (DSST) Algorithm	Fitting a quadratic surface to the 3D road point cloud and comparing it with the actual 3D road point cloud extracts the pothole point cloud. The DSST algorithm is then used to detect the potholes.	Accuracy: 98.7%
Ma et al. (2023) [128]	Mobile laser scanning data	Directed distance, skew distribution, density clustering	Combining directed distance calculation with density clustering for the singularization and denoising of potholes, potholes are detected using the negative skew distribution and skewness coefficient of the directed distance histogram.	Accuracy: 91% Recall: 82%
Fan and Chen (2023) [129]	Mobile laser scanning data	PointNet, PointCNN, region-growing algorithm	Based on MLS point cloud data, a comparison was conducted between deep learning algorithms and the region-growing algorithm.	Recall: 89.2% IoU: 0.82
Sun et al. (2025) [130]	3D point cloud	Voxel filtering, RANSAC, Euclidean clustering, Alpha Shapes algorithm	Voxel filtering is used for noise reduction, RANSAC and Euclidean clustering for point cloud segmentation, and the Alpha Shapes algorithm for 3D reconstruction to detect potholes and estimate their volumes.	Accuracy: 96.4%

**Table 6:** Review of studies employing machine learning/deep learning methods.

Authors	Date	Method	Descriptions	Performance
Shah and Deshmukh (2019) [138]	Color image	ResNet-50, YOLO	Using the ResNet-50 network to classify normal roads, speed bumps, and potholes from images achieves a true positive rate (TPR) of 88.9%.	True positive: 88.9%
Arunpriyan et al. (2020) [140]	Color image	Data augmentation, SegNet	SegNet, a semantic segmentation deep CNN, has 91.781% global accuracy but performs poorly in detecting unmarked speed bumps and vertical-view images.	Accuracy: 91.781% MIoU: 48.872
Gupta et al. (2020) [141]	Thermal image	ResNet34-SSD, ResNet50-RetinaNet	Innovatively combining thermal images with deep learning for pothole detection, the improved ResNet50-RetinaNet achieves 91.15% average precision.	Accuracy: 91.15%
Dewangan and Sahu (2020) [143]	Color image	Self-built CNN, distance estimation algorithm based on pixel changes	A deep learning and computer vision-based speed bump detection model is proposed, achieving 98.54% accuracy, 99.05% precision, and 97.89% F1-score in real-time scenarios.	Accuracy: 98.54% Precision: 99.05% F1-score: 97.89%
Fan et al. (2021) [144]	RGB image, disparity image, transformed disparity image	SoAT CNNs, GAL-DeepLabv3+	The first stereo vision-based road pothole monitoring dataset and a new disparity transformation algorithm have been released. The GAL-DeepLabv3+ model achieves the highest overall detection accuracy across all data modalities.	Precision: 89.819% Accuracy: 98.669% Recall: 83.205% F1-score: 89.802%
Mohan and Sriharipriya (2022) [145]	Color image	YOLOX-Nano	A pioneering study introduces the first application of the YOLOX object detection model to pothole detection, achieving 85.6% average precision with the lightweight YOLOX-Nano variant, which occupies only 7.22 MB of storage.	Precision: 85.6% Size: 7.22 MB
Aishwarya et al. (2023) [148]	Color image	Faster R-CNN, YOLOv5, Negative Sample Training (NST)	State-of-the-art Faster R-CNN and YOLOv5 models were used, with the negative sample training (NST) method enhancing detection accuracy—achieving 5.58% and 2.3% increases for significant speed bumps, respectively.	Accuracy: 98.8% Size: 42.2 MB
Hussein et al. (2024) [150]	Color image	Data augmentation, YOLOv8	The YOLOv8n model performs best in detecting both labeled and unlabeled speed bumps, with an average precision (mAP) of 0.81. This method integrates a Kinect Xbox camera on the Jetson Nano developer kit to enable distance estimation for detected speed bumps.	Precision: 82% Recall: 79%
Bodake and Meeeshala (2025) [152]	Color image	FTayCO-DCN, Panoramic Image Conversion, Grayscale Conversion	The integrated framework combining the improved FTayCO algorithm and the DCN classification model achieves pothole detection with 99.06% accuracy, 99.09% sensitivity, and 99.04% specificity.	Accuracy: 99.06% Sensitivity: 99.09% Specificity: 98.33%
Chandak et al. (2024) [153]	Color image	YOLOv4, SSD-MobileNet, data augmentation	A comparative analysis of YOLOv4 and SSD-MobileNet reveals that SSD-MobileNet exhibits superior performance in terms of accuracy (0.42), recall (0.81), and F1-score (0.82). Additionally, SSD-MobileNet outperforms YOLOv4 in inference efficiency, with an inference time of 7 ms compared to 52.51 ms for YOLOv4.	Precision: 85.48% Recall: 81% F1-score: 82%

**Table 7:** Review of studies using multi-sensor fusion methods.

Authors	Date	Method	Descriptions	Performance
Joubert et al. (2011) [156]	3D point cloud, color image	Random Sample Consensus algorithm, contour detection	Integrates 3D point clouds with high-speed USB-captured images; uses RANSAC to separate road surface from depression point clouds and edge detection to calculate depression width/depth.	/
Li et al. (2016) [159]	2D images, GPR data	Image processing, based on geometric active contour model	Integrates 2D images and GPR data; estimates pothole location/size from GPR data, maps them to image.	Accuracy: 88% Recall: 90% Precision: 94.7%
Kang and Chio (2017) [160]	2D radar point cloud, color image	Median filtering, Gaussian blurring algorithm, Canny edge detection	2D LiDAR acquires road distance/angle information; through noise filtering, clustering, line extraction, and data gradient analysis, obtains pothole contours.	/
Yun et al. (2019) [161]	2D images, 3D point clouds	Image binarization, Gaussian filtering, median filtering, Harr, HOG, SVM	Uses two detectors to extract/verify speed bumps: extracts speed bump candidate regions via image patterns; detects speed bump area/height via point cloud data, HOG, and SVM.	Precision: 88% Recall: 95% F1-score: 91%
Salaudeen and Celebi (2022) [162]	Color Image (Low Resolution and Super-Resolution)	ESRGAN, YOLOv5, EfficientDet	Super-resolution reconstruction of low-resolution road images at 4× scale is performed using ESRGAN. Subsequently, two object detection models, YOLOv5 and EfficientDet, are employed for training and inference on the enhanced high-resolution images.	Precision: 97.6% Recall: 70%
Roman-Garay (2025) [163]	2D images, 3D point clouds	Segformer, RANSAC, Fuzzy Logic Model	Creates dataset with 2D images/3D point clouds; uses transfer learning (Segformer) to achieve 90.87% recall, 90.01% accuracy, 90.43% F1 score.	Accuracy: 90.01% Recall: 90.87% F1-score: 90.43%

**Table 8:** Classification of various detection methods.

Method	Advantages	Disadvantages
Traditional dynamics	Easy to deploy, low cost	Poor real-time performance
2D Image Processing	Simplicity, low cost	Sensitive to lighting conditions and road conditions
3D Point Cloud Analysis	Detailed surface information	Computational complexity, high equipment costs
Machine/Deep Learning	High detection accuracy, high robustness, high reliability	Requires a large amount of training data
Multi-sensor fusion methods	Higher accuracy, robustness	Increased complexity, integration challenges

**Table 9:** Based on the average precision, recall, F1 score and computational cost of each detection method as determined by the review study results.

Method	Precision	Recall	F1-Score	Computational Cost
Traditional dynamics	85%	/	/	Low
2D Image Processing	76%	69%	70%	Low
3D Point Cloud Analysis	85%	84%	83%	High
Machine/Deep Learning	88%	86%	87%	Medium
Multi-sensor fusion methods	91%	87%	90%	High

**Table 10:** Reviewed the studies that can provide elevation information.

Authors	Date	Method	Descriptions
Chitale et al. (2020) [169]	Color image	YOLOv3, YOLOv4, Triangular Similarity Based on Image Processing	The YOLOv4 model exhibits excellent performance, with an mAP of 0.933 and IoU of 0.741. Combined with the triangular similarity principle, pothole depth can be estimated, with an error of 5.868%.
Ahmed et al. (2021) [170]	Dynamic image	High-pass filtering, Gaussian filtering, SIFT feature matching, laser triangulation measurement	Using SIFT feature matching and the 5-point algorithm, a 3D point cloud is generated. Under static imaging conditions, the average depth and perimeter errors are 5.3% and 5.2%, respectively.
Das and Kale (2021) [171]	Live video	CNN, RPN, frequency heatmap calibration	The method combines CNN and RPN, converting real-time video into a VIBGYOR color scale heatmap via frequency heatmap calibration to enhance system adaptability to complex environments.
Li et al. (2022) [172]	Stereo image	Binocular stereo vision, three-dimensional reconstruction, genetic algorithm	Combining binocular stereo vision, 3D pothole features are extracted using point cloud interpolation and plane fitting algorithms, achieving depth and area detection with average accuracies of 98.9% and 98%, respectively.
Wang et al. (2023) [78]	Multi-view 2D images	PP-SFM algorithm, Trans-3DSeg	An approach based on PP-SFM generates 3D point clouds, with the Trans-3DSeg model for training. Under low-light conditions, segmentation accuracy is 91.86% and F1 score is 92.13%.
Ranyal et al. (2023) [173]	2D image	Improving CNN architecture–RetinanNet, motion structure photogrammetry technology	An intelligent pavement detection system employs the improved CNN RetinanNet. It uses motion structure photogrammetry to simulate pothole 3D point clouds, achieving an F1 score up to 98% on the dataset with average pothole depth estimation error below 5%.
Singh et al. (2024) [174]	Color image	Faster RCNN Resnet 50 FPN	The Faster R-CNN ResNet-50 FPN model achieves training and validation accuracies of 96% and 85%, respectively. Using image processing and ultrasonic sensors, pothole depth can be estimated.
Park and Nguyan (2025) [175]	Color image	YOLOv8-seg, optical geometric principles	Using binocular vision, the YOLOv8 instance segmentation model, and optical geometry principles, real-time pothole area and depth are calculated with average error below 5%.

## Data Availability

No new data were created or analyzed in this study. Data sharing is not applicable to this article.

## Abbreviations

The following abbreviations are used in this manuscript:

2D	Two-dimensional
3D	Three-dimensional
FFT	Fast Fourier transform
CNN	Convolutional Neural Network
ICV	Intelligent connected vehicle
YOLO	You Only Look Once
ToF	Time of flight
MLS	Mobile laser scanning
SIFT	Scale-invariant feature transform
GNSS	Global Navigation Satellite System
SVM	Support Vector Machine
FIS	Fuzzy inference system
AUC	Area under Curve
CWT	Continuous wavelet transform
IMU	Inertial Measurement Unit
EFDD	Extract data features filter
CNN-LSTM	Convolutional Neural Network- Long Short-Term Memory
ROI	Region of interest
FCM	Fuzzy c-means
GMM	Gaussian Mixture Model
MRF	Markov Random Field
RANSAC	Random Sample Consensus
IoU	Intersection over Union
SFCW	Stepped-frequency continuous wave
DSST	Discriminative scale space tracking
mAP	Mean Average Precision
FNR	False negative rate
TPR	True positive rate
HOG	Histogram of Oriented Gradients
NST	Negative sample training
SPP	Spatial pyramid pooling
FPN	Feature pyramid network
GPR	Ground-penetrating radar
GPS	Global positioning system
RMSE	Root mean square error
CoU	Complete-IoU
DIoU	Distance-IoU
SFM	Structure from motion
RPN	Region proposal network
DR	Damage rate
PCI	Pavement condition index
ADAS	Advanced driver assistance systems
NAS	Neural architecture search
KD	Knowledge distillation
3F2N	Three-Filters-To-Normal
DBSCAN	Density-Based Spatial Clustering of Applications with Noise
cGAN	Conditional generative adversarial networks
GAL	Graph Attention Layer
AP	Average precision
DCN	Deep convolutional neural networks
ESRGAN	Enhanced Super-Resolution Generative Adversarial Network
LR	Low-resolution
SR	super-resolution
